# Diagnosis of Wilson Disease and Its Phenotypes by Using Artificial Intelligence

**DOI:** 10.3390/biom11081243

**Published:** 2021-08-20

**Authors:** Valentina Medici, Anna Czlonkowska, Tomasz Litwin, Cecilia Giulivi

**Affiliations:** 1 Department of Internal Medicine, Division of Gastroenterology and Hepatology, University of California Davis, Sacramento, CA 95817, USA; vmedici@ucdavis.edu; 2 Second Department of Neurology, Institute of Psychiatry and Neurology, 02-957 Warsaw, Poland; czlonkow@ipin.edu.pl (A.C.); tomlit@medprakt.pl (T.L.); 3 Department of Molecular Biosciences, School of Veterinary Medicine, University of California Davis, Davis, CA 95616, USA; 4 Medical Investigation of Neurodevelopmental Disorders (MIND) Institute, School of Medicine, University of California Davis, Sacramento, CA 95817, USA

**Keywords:** Wilson disease, copper, mitochondria, liver, intermediary metabolism, urea cycle, amino acids, Krebs’ cycle, artificial neural network, diagnosis prediction

## Abstract

WD is caused by *ATP7B* variants disrupting copper efflux resulting in excessive copper accumulation mainly in liver and brain. The diagnosis of WD is challenged by its variable clinical course, onset, morbidity, and *ATP7B* variant type. Currently it is diagnosed by a combination of clinical symptoms/signs, aberrant copper metabolism parameters (e.g., low ceruloplasmin serum levels and high urinary and hepatic copper concentrations), and genetic evidence of *ATP7B* mutations when available. As early diagnosis and treatment are key to favorable outcomes, it is critical to identify subjects before the onset of overtly detrimental clinical manifestations. To this end, we sought to improve WD diagnosis using artificial neural network algorithms (part of artificial intelligence) by integrating available clinical and molecular parameters. Surprisingly, WD diagnosis was based on plasma levels of glutamate, asparagine, taurine, and Fischer’s ratio. As these amino acids are linked to the urea–Krebs’ cycles, our study not only underscores the central role of hepatic mitochondria in WD pathology but also that most WD patients have underlying hepatic dysfunction. Our study provides novel evidence that artificial intelligence utilized for integrated analysis for WD may result in earlier diagnosis and mechanistically relevant treatments for patients with WD.

## 1. Introduction

Wilson disease (OMIM 277900) is caused by homozygous or compound heterozygous variants affecting the *ATP7B* gene (OMIM 606882) on chromosome 13q14. This gene encodes for a polypeptide that, when acting as a dimer, exhibits a plasma membrane copper-transport activity [1,2]. The protein has several membrane-spanning domains, an ATPase consensus sequence, a hinge domain, a phosphorylation site, and at least two putative copper-binding sites located mainly at the Golgi apparatus. By functioning as a monomer, it exports copper out of the cells, guaranteeing the efflux of hepatic copper into the bile. Alternate transcriptional splice variants encoding different isoforms with distinct cellular localizations have been characterized.

Challenges to WD diagnosis are complicated by two factors: the type of *ATP7B* variant and the clinical course of the disease. Most of the *ATP7B* gene variants observed in patients include nonsense and frameshift mutations along with deletions, but few of the truncated or modified ATP7B proteins still conserve some of the native activity. This might constitute the main reason underlying the inconclusive attempts to correlate genotype with phenotype [3,4] when including copper parameters [5]. WD presents with a variable clinical course. For instance, young adults more frequently manifest the first symptoms of WD [6,7], but some patients present a late onset of the disease while others may not show overt signs of copper toxicity at all. In addition, WD is usually classified into three phenotypes defined as primarily hepatic (40%), neurological (40%), and psychiatric or asymptomatic (20%). However, this classification is not exactly fine-tuned to the patients’ presentation of WD, as neurological signs (e.g., tremor, ataxia, dystonia, and parkinsonism) are often observed concomitantly with hepatic metabolic defects [6,7,8,9,10]. Furthermore, neurological signs usually present later than those associated with liver pathology [7,9,10,11]. These challenges at recognizing the signs of WD may hamper clinicians’ ability to make an accurate diagnosis, impacting the delivery of personalized treatments that may minimize the progression of the disease.

Considering the above challenges, the aim of this study was to apply artificial intelligence to aid in the diagnostic process of WD and its hepatic or neurological manifestations. In this regard, a neural network is a simulation of a biological brain (a.k.a. Artificial Neural Network or ANN) and a branch of artificial intelligence. ANN is first “trained” by having it process several input patterns and showing what output resulted from each input pattern. Once trained, the ANN can recognize similarities when presented with a new input pattern, resulting in a predicted output pattern. As such, ANN detects early warning signals of critical transitions defined as sudden and large-scale state transitions that occur in complex systems [12,13]. In the case of WD, we can speculate that ANN could contribute both to the early diagnosis of the disease and can help characterize and predict the disease phenotypes. Here, we will use the breadth of clinical and molecular outcomes to detect input similarities, thereby allowing ANN to build a predictive model for WD phenotypes by considering the diagnosis of WD and its phenotypes as critical transition points [12,14,15,16,17,18,19]. This can be achieved even with relatively higher inter-subject variability as observed in humans compared to isogenic/cloned WD animal models maintained under rigorous, controlled conditions.

## 2. Materials and Methods

### 2.1. Biological Samples

The patients’ demographic and clinical data (age, sex, and BMI; WD diagnosis and subclassification into hepatic, neurological or asymptomatic [5]) were collected at a single center, the 2nd Department of Neurology, Institute of Psychiatry and Neurology in Warsaw, Poland. Patients were all recruited when pre-treatment (i.e., not receiving any anti copper treatment). All patients were diagnosed with WD based on the Leipzig criteria, including low ceruloplasmin levels, increased 24 h urinary and hepatic copper levels, presence of Kayser–Fleischer rings, presence of neurological symptoms, Coombs-negative hemolytic anemia, and eventual genetic testing results if available, as previously described [20,21]. Other basic laboratory liver tests (i.e., total bilirubin) were performed in the hospital laboratory by using standard methods. Samples from control healthy subjects were obtained from the same community in Poland. The study was approved by the local bioethics committee and all patients provided written informed consent prior to participation following the Declaration of Helsinki.

Mitochondrial DNA (mtDNA) copy number and deletions were determined in whole blood by following the previously described protocol [22]. Of note, the subjects included in the present analysis are the same subjects described in our previous mtDNA study [22]. Serum metabolomics were determined by mass spectrometry as described before [23,24]. Serum metabolites were normalized to the average of control values and expressed as log2 fold change (log2 FC). The missing values were replaced by using the k-nearest neighbor algorithm feature approximation.

### 2.2. Artificial Neural Network (ANN) for Diagnosis

The neural-network design for the SFAM algorithm consisted of a three-layer network: an input layer, with four units for the diagnostic criteria defined as control, asymptomatic WD or WDA, hepatic WD or WDH, and neurologic WD or WDN; sex, age, and BMI, 66 units for metabolites relevant to liver metabolism, 3 units for metabolite ratios, and 2 units for mtDNA-associated data; a hidden layer, with 11 units; and an output layer for the diagnosis of WD. A NeuNet Pro software (CorMac Technologies Inc., Thunder Bay, ON, Canada), SFAM algorithm was used for the prediction of WD and its phenotypes. The unsupervised training variables included all of those indicated above. In order to select the size of the training set, we performed a learning curve analysis [25]. Essentially, we withdrew a relatively small random sample from our data to train an ANN and used this training set to predict a randomly sampled test set. Then, the size of our training sample was iteratively increased while keeping the same test set. By tracking the degree to which predictive accuracy on the fixed test set increased with the training set size, we obtained the training data that were needed until the differences between the network classifications and the clinical diagnoses became acceptable (41 training samples and 21 test ones). We also repeated the learning curve analysis multiple times with different test set samples to further reduce variation in predictive accuracy. Finally, the patterns of input facts associated with diagnoses were trained with randomly selected 41 subjects from the set 62 with known clinical status. Once the network was trained, the remaining 21 subjects were “tested” by means of the trained network. The neural network classifications were then compared with the known clinical diagnoses to observe whether the network was able to classify the disease status with reliability. The same data were analyzed with the Visual Rule Extraction, which is a highly optimized version of the C4.5 algorithm published by J. Ross Quinlan [26] for generating a decision tree with adjustable tree pruning. Inductive Rule Extraction, which is related to the fields of machine learning, knowledge discovery, expert systems, and artificial intelligence, is often called “Decision Tree Classification”. The method depends on the concept of entropy, which was introduced in the field of information theory by Dr. Shannon >70 years ago [27]. Our analysis included a 75% pruning with 4 minimum subjects.

## 3. Results and Discussion

A total of 62 subjects were included in our analysis (Table S1). Of those, 47 were diagnosed with WD (23 females/24 males) with prevalent hepatic (WDH; *n* = 18; 11 females/7 males) and prevalent neurological (WDN; *n* = 18; 7 females/11 males) manifestations or were asymptomatic (WDA; *n* = 11; 5 males/6 females). The average age of the 15 healthy control subjects (10 females/5 males) was 36 ± 9 years (mean ± SD), which is not different from that of patients with WD (34 ± 11 years).

In order to apply ANN, we used two-thirds of the randomly selected samples (from all 62) to train the ANN by utilizing as input data the diagnosis of WD (healthy control, WDA, WDH, and WDN), age, sex, BMI, total bilirubin, ceruloplasmin levels, mtDNA copy number and deletions from whole blood, and 66 serum metabolites relevant to liver physiology, all evaluated by mass spectrometry. We also included the following three relevant metabolite ratios: lactate-to-pyruvate, as a feature of mitochondrial dysfunction [28]; cystine levels normalized to the sum of cystine and cysteine, as a marker of oxidative stress [29,30]; and the Fischer’s ratio (the ratio of branched chain amino acids (BCAA) to that of aromatic amino acids (AAA) [31]), as a biomarker of advanced fibrosis (Table S1).

**Figure 1:**
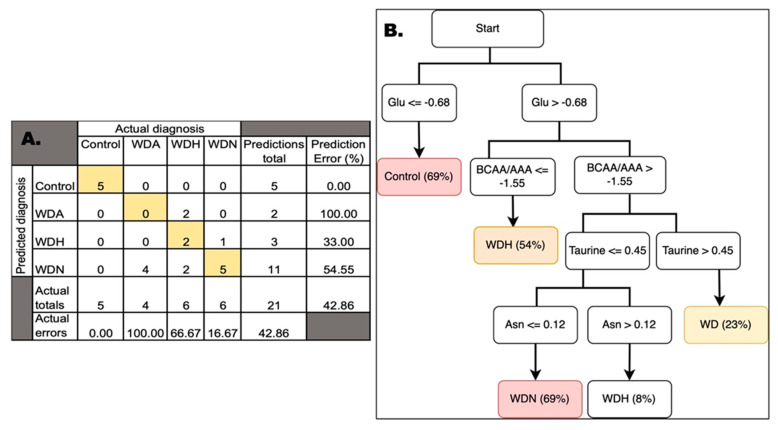
Confusion matrix and decision tree for the diagnosis of WD and its manifestations by using artificial neural network. (**A**) The testing samples (*n* = 21) were “trained” by using the trained ANN obtained with the training set. By applying a simplified fuzzy adaptive resonance theory map, the top part of the table shows the actual diagnosis vs. the predicted one. Numbers in boxes represent the number of subjects except for errors which are expressed in percentages. Colored boxes are those matching the actual and predicted diagnoses. (**B**) Decision tree showing the main outcomes needed to be tested in order to reach the diagnosis of WD and its phenotypes. Diagnosis confidences (in percentage) are stated between parentheses. Other details are found in the text.

By utilizing a simplified fuzzy adaptive resonance theory map to predict a class (in our case, WD diagnosis), the ANN classification identified subjects with WD vs. those without it with an accuracy of 100% (95% CI = 83.89 to 100%) and sensitivity and specificity of 100% (95% CI, respectively, 79.41% to 100% and 47.82% to 100%). Overall, when the different phenotypes are considered, the ANN showed an accuracy of 57.14% (Figure 1A; Table S2). The control and WDN diagnoses had the least errors (0% and 16.67%, respectively), whereas WDA and WDN diagnoses had 100% and 66.67% errors. The mismatches between predicted and the actual diagnoses were mostly the result of the reclassification of 60% of WD-affected subjects to the WDN diagnosis (all four WDA and two of the six diagnosed as WDH), suggesting the possible occurrence of undetected or subclinical neurological issues in WD patients diagnosed as either WDA or WDH.

The same set was analyzed by using a Visual Rule Extraction algorithm to ascertain what outcomes needed to be collected and in what order for reaching a reliable diagnosis, which patients need a second opinion regarding their diagnosis, and finally what combination of factors are important in reaching a diagnosis (Figure 1B; Table S3). Our results show that the resulting flow chart for determining diagnosis was built basically on values from nine amino acids and derivatives (glutamate, asparagine, taurine, branched-chain, and aromatic amino acids; Figure 1B). Notably, advanced liver disease is associated with metabolic derangements, especially for amino acid levels, and most of these amino acids are directly or indirectly associated with the urea (nitrogen disposal) and Krebs’ cycles. These results also highlight the critical role of hepatic mitochondria in WD morbidity as our own previous studies showed [22,32].

**Figure 2:**
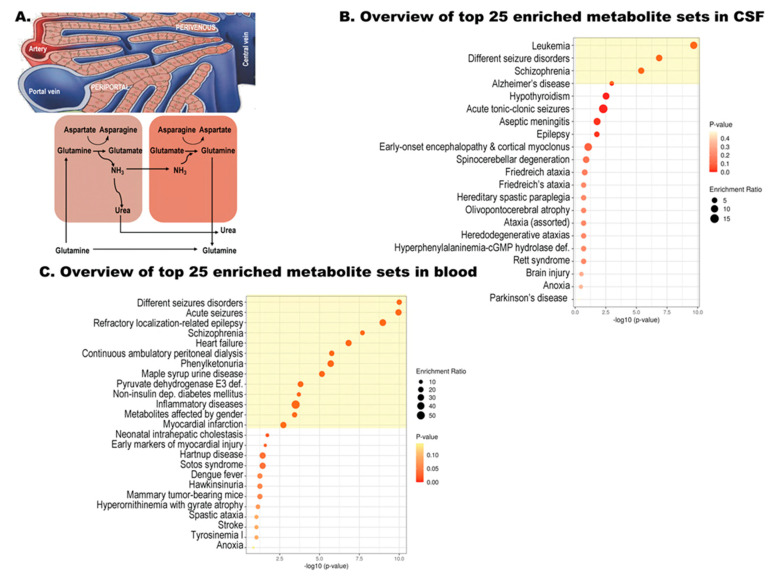
Enrichment analysis of outcomes identified by ANN and the intercellular glutamine cycle in WD. (**A**) Diagram showing the intercellular glutamine cycle as it occurs between the periportal cells and the perivenous ones surrounding the central vein. Copper accumulation induces liver toxicity, which results in lower urea cycle function undermining a safe disposal of the excess of ammonia as urea. Excess of ammonia, formed by the action of glutaminase on glutamine (among others), is then used to generate glutamate at the expense of the Krebs’ cycle intermediate alpha-ketoglutarate as well as increases in asparagine at the expense of aspartate. The lower activity of the Krebs’ cycle results in lower ATP production, which may challenge the ATP-driven generation of glutamine from glutamate. Enrichment analyses were performed by using the amino acids and derivatives identified under Figure 1B as input against a disease signature database in CSF (**B**) and blood (**C**). Those highlighted in yellow had an FDR < 0.05. The analysis was performed by using MetaboAnalyst [33].

The healthy control subgroup was identified with a 69% confidence by considering only the levels of the amino acid glutamate (log2FC ≤ −0.68; Figure 1B). In patients with liver disease, as the hepatocytes cannot convert ammonia into urea and glutamine rapidly enough, the blood ammonia level rises. Shunting of blood from the liver—as observed in portal hypertension with interference with the intercellular glutamine cycle (Figure 2A)—results in increased ammonia as well as glutamate levels. These changes are the likely underlying metabolic explanation of our findings, indicating that glutamate levels could be a discriminating factor between healthy controls and patients with WD.

With high glutamate levels, low BCAA/AAA ratio (log2FC ≤ −1.55) indicates a diagnosis of WDH with 54% confidence. If the levels of the BCAA/AAA are above the threshold, then low levels of both taurine (log2FC ≤ 0.45) and asparagine (log2FC ≤ 0.12) result in a diagnosis of WDN with a 69% confidence (Figure 1B). If the levels of asparagine are above the threshold, the diagnosis is WDH with an 8% confidence. Although most amino acids are metabolized by the liver, BCAAs are metabolized exclusively by skeletal muscle. Hence in progressive liver failure and end stage liver disease, as observed in association with copper accumulation, the latter amino acids’ metabolism remains unaffected, whereas the metabolism of other amino acids, especially the aromatic ones, is severely impaired. Therefore, blood concentrations of BCAA are normal, whereas those of AAA increased, thereby increasing the BCAA/AAA ratio. Since both types of amino acids are transported into the brain via the same carrier, the change in concentration ratio increases the amount of AAA that enters the brain. Considering that these amino acids (tyrosine and tryptophan) are the precursors of crucial biogenic amines, dopamine, noradrenaline, and serotonin (5-hydroxytryptamine) and given that that the serotonin synthesis in the brain does not contain a flux-generating step, these changes in amino acid blood levels increase the concentrations of amines in the brain. As serotonin levels in brain promote sleep, large amounts of tryptophan and serotonin could explain the neurological issues and lethargy observed in some WD-affected patients [34]. These imbalances in neurotransmitters along with deficits in the disposal of nitrogen via the urea cycle and the siphoning off alpha-ketoglutarate from Krebs’ cycle may further contribute to energy failure and ammonia toxicity in the CNS.

The combination of high glutamate and BCAA/AAA ratio with levels of taurine above the threshold results in WD diagnosis with a 23% confidence (Figure 1B). High levels of taurine somehow seem to protect WD-affected patients from entering a path that results in WDN diagnosis. Taurine is an abundant intracellular free amino acid that has a central role in brain development, and it is the second most important inhibitory neurotransmitter after GABA. It also forms conjugates with bile acids and may enhance bile flow and increase cholesterol clearance by the liver. Taurine, in the context of copper-induced oxidative stress, may also play a role in salvaging toxic intermediates (see [35] and references therein). Interestingly, taurine in adults can be obtained from the diet or from synthesis from cysteine when vitamin B6 is present. Although vitamin B6 deficiency does not appear to be frequently associated with new penicillamine formulations, it has been proposed that some of the side effects of copper chelator D-penicillamine may be the result of interference with vitamin B6 metabolism, thereby promoting neurological issues [36,37]. Since the studied patients were not receiving any treatment at the time of the blood draw, it is tempting to propose that copper-mediated increases in oxidative stress may reduce cysteine levels as well as those of B6, resulting in lower taurine which then triggers some of the neurological symptoms due to an imbalance between excitatory and inhibitory neurotransmitters.

The decision tree showed some phenotypes (control, WDH, and WDN) that were diagnosed with suitable confidence levels, whereas it was less useful for WDA. This suggests that, according to the ANN analysis, most subjects affected by WD could actually be either WDN or WDH even when they are still clinically asymptomatic. Notably, when taurine and asparagine levels are low and glutamate is high, increases in the BCAA/AAA ratio shifts the diagnosis from WDH to WDN suggesting that these parameters should be tested in all WD patients in order to monitor their clinical manifestations and possible progression from hepatic to neurologic signs and symptoms.

Enrichment analysis performed with the nine amino acids and derivatives implicated in the diagnosis of WD (from Figure 1B) against a database of disease signatures in human cerebral spinal fluid (Figure 2B) and blood (Figure 2C) indicated a significant overlap with manifestations associated with WD. Among them, seizures, ataxia, inflammation, amino acid or metabolic disorders (overlapping with diabetes, Hartnup, and Tyrosinemia), systemic disorders (overlapping with Friedreich’s ataxia), cognitive impairment [38], and cholestasis [39] were identified.

**Figure 3:**
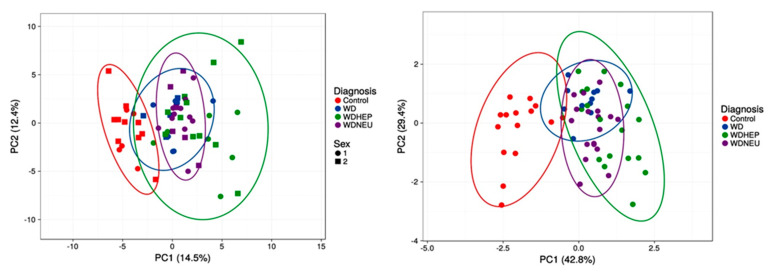
Principal component analysis of the four diagnostic groups based on all outcomes and the selected nine amino acid and derivatives from the decision tree. PCA (utilizing ClustVis 2.0 [40]) was applied to the test dataset by utilizing all outcomes as input (top panel; *n* = 62 points). Unit variance scaling was applied to rows; singular value decomposition with imputation was used to calculate principal component analysis (to both top and bottom panels). Other options were set as it follows: no transformation of data was performed, and no collapse of columns with similar annotations was performed; maximum percentage of unavailable data allowed in both rows and columns was set at 99.99; row centering; no removal of constant columns; row scaling was based on unit variance scaling, and the PCA method was calculated by using singular value decomposition. The *x*-axis and *y*-axis show principal component 1 and principal component 2 that explain 14.5% and 12.4% of the total variance, respectively. Prediction ellipses possess 0.95 probability, and a new observation from the same group will fall inside the ellipse (both panels). All other data including PCA variances and loadings are summarized under Tables S4 and S5, respectively. Bottom panel was performed by using only the BCAA/AAA ratio, glutamate, asparagine, and taurine levels. The *x*-axis and *y*-axis show principal component 1 and principal component 2 that explain 42.8% and 29.4% of the total variance, respectively. All other data including PCA variances and loadings are summarized under Tables S6 and S7, respectively.

Finally, principal component analysis performed with all outcomes vs. the nine amino acids and derivatives from the decision tree showed that the separation between healthy controls and WD patients is the most efficient. Conversely, both WDA and WDN diagnoses seem to be subsets of the larger WDH group, suggesting that all patients may have various degrees of underlying and potentially underdiagnosed or underrecognized hepatic function impairment (Figure 3).

## 4. Conclusions

By evaluating nine amino acids and derivatives, it is possible to diagnose WD with an acceptable level of confidence for those having hepatic and neurological manifestations. Surprisingly, the outcomes usually tested in WD, such as ceruloplasmin and total bilirubin levels or common demographic and clinical parameters including age, sex, or BMI, did not play any role in our decision tree. The finding that nine amino acids reflect the WDN and WDHmainly liver function as well as the interaction between the urea and the Krebs’ cycle is consistent with the role of mitochondrial dysfunction in both patients and mouse models of the disease [22,32,41,42,43,44,45,46,47,48,49,50,51,52,53,54,55,56,57]. One major limitation of the studied cohort is the lack of direct (liver biopsy and histological analyses) or indirect assessments (liver and brain imaging) of disease morbidity. In addition, we are not comparing WD cases to other etiologies of liver diseases, and we do not have a prospective cohort to assess the longitudinal risks in developing WD manifestations. Some of our findings may not be specific of WD but could be associated with liver fibrosis and portal hypertension in general. However, the proposed algorithm could have incremental value if added to existing diagnostic parameters of altered copper metabolism (except ceruloplasmin) or findings from liver or brain imaging [58], and it could streamline the diagnostic process. It could be argued that adding histological reports may improve the accuracy of the ANN-based algorithm. However, non-invasive diagnoses and assessments of liver disease are becoming the standard-of-care for most liver diseases. As such, access to histology reports is less common. A scoring system based on liver histology will not likely help current and future clinical practice. On the other hand, future studies should attempt an integration of validated WD scoring systems, including the Leipzig score, to ANN approaches in order to further improve their clinical accuracy.

Furthermore, our findings may have relevance when designing targeted therapies or when optimizing dietary approaches in the management of patients with WD. Dietary approaches in WD should aim at reducing the urea cycle overload and, consequently, mitochondrial dysfunction, while assuring adequate protein intake in order to minimize sarcopenia associated with portal hypertension. In addition, the adjustment of the BCAA/AAA ratio, which has been extensively studied as an approach to the treatment of hepatic encephalopathy [59,60,61,62,63,64,65], may be particularly helpful in WD when hepatic and neurological manifestations coexist.

Ultimately, ANN represents a future option when considering the diagnosis of WD, especially in those frequent cases with uncertain clinical presentation, and offers the opportunity for therapeutic improvements. 

## Data Availability

The metabolomic data presented in this study were published before [22,23,24]. No new metabolomic data were created in this study.

## Supplementary Materials

The following are available online at https://www.mdpi.com/article/10.3390/biom11081243/s1, Table S1: Demographics, clinical, and molecular outcomes utilized in this study, Table S2: Results from SFAM algorithm, Table S3: Results from the visual rule extraction algorithm, Table S4: PCA variance when using all outcomes, Table S5: PCA loadings when using all outcomes, Table S6: PCA variance when using the outcomes selected by the visual extraction rule, Table S7: PCA loadings when using outcomes selected by the visual extraction rule algorithm.
