# Supplementary material for: Diagnosis of Wilson Disease and Its Phenotypes by Using Artificial Intelligence
Source: Biomolecules. 2021 Aug 20;11(8):1243. doi: 10.3390/biom11081243 (PMC8394607; doi:10.3390/biom11081243)
Supplement: Supplementary file 1 [file biomolecules-11-01243-s001.zip › biomolecules-1338897-supplementary.pdf]

Supplemmentary Table S1. Demographics, clinical and molecular outcomes utilized in this study

Subject ID	Diagnosis	Sex	Age	BMI	mtDNA CN	mtDNA deletions	L/P	BCAA/AAA	Ceruloplasmin	Total bilirubin	CysCys/Total Cys	Pyruvic acid	L-Glutamic acid	Oxoglutaric acid
M5No_WD	Control	Male	32	-0.6	0.3	0.67	0.37	1.29	0.98	-0.45	-1.76	-0.33	-1.73	-1.55
M1No_WD	Control	Male	27	-0.4	0.95	-0.31	-0.41	1.7	0.83	0.14	-1.5	0.01	-1.5	-1.33
M4No_WD	Control	Male	33	0.6	1.6	0.6	-1.52	1	1.7	0.38	1.08	1.64	-2.09	-0.33
M3No_WD	Control	Male	28	1.1	1.37	0.47	-1.44	1.4	1.33	0.54	0.97	1.03	-1.75	-0.8
M2No_WD	Control	Male	45	1.53	0.79	0.06	0.12	1.27	1.17	-1.42	-1.27	0.46	-0.08	-0.33
K2No_WD	Control	Female	26	-1.57	-0.12	-0.13	0.76	0.72	1.17	0.4	-1	-0.36	-0.68	-1.21
K3No_WD	Control	Female	49	0.55	1.54	-0.25	0.54	0.93	0.53	0.33	-0.7	-0.23	-1.19	-0.93
K9No_WD	Control	Female	52	-0.03	0.49	-0.94	-2.04	-0.31	0.22	-0.15	-1.18	1.55	-1.14	-0.37
K7No_WD	Control	Female	34	-1.31	0.38	-1.51	0.96	0.78	1.56	-0.83	0.38	-0.8	-1.95	-1.25
K5No_WD	Control	Female	30	-1.56	1.29	-0.53	-0.28	1.19	1.14	-2.68	-1.24	-0.45	-1.9	-2.1
K8No_WD	Control	Female	27	-0.96	-0.63	-1.18	1.11	1.54	1.24	0.29	-0.5	-1.59	-1.94	-0.55
K10No_WD	Control	Female	52	-0.51	0.71	1.09	-0.19	1.42	1.45	-0.2	-1.39	0.92	-0.33	-0.77
K6No_WD	Control	Female	38	-1.62	0.98	-0.58	-1.48	1.41	1.06	-1.08	-1.09	1	-1.84	-0.43
K1No_WD	Control	Female	38	-1.16	0.72	-0.34	-0.38	0.96	0.69	-0.62	0.79	0.59	-2.02	-0.72
K4No_WD	Control	Female	31	-0.39	0.26	-1.02	-0.84	1.13	0.89	-0.32	0.45	0.69	-1.86	-0.74
1506WD	WD	Male	41	0.52	-2.92	-0.19	-0.77	-0.79	-0.96	-0.64	-1.47	0.88	1.05	1.35
1593WD	WD	Male	25	-0.49	-1.49	-0.77	-0.06	0.92	0.24	-0.49	-1.46	-0.34	0.21	0.63
1784WD	WD	Male	39	1.42	0.08	0.17	-0.07	1.04	0	0.55	-2.15	1.82	-0.18	0.53
1986WD	WD	Male	52	0.94	-0.13	1.02	0.51	-0.23	0.1	-0.08	-0.02	-0.54	0.45	0.47
2486WD	WD	Male	25	-0.49	-0.52	-0.03	-0.7	0.33	1.25	-0.44	0.78	0.46	1.18	2.2
2251WD	WD	Male	21	1.09	0.47	1.18	0.51	0.35	-0.86	-0.88	0.48	-0.38	0.81	-1.02
2055WD	WD	Female	20	0.36	-0.27	0.06	-0.13	-0.45	0.4	-0.19	-0.21	0.48	0.1	0.94
2124WD	WD	Female	52	-0.4	-0.24	0.43	-0.81	0.19	0.42	-1.07	0.69	0.33	0.27	0.04
2275WD	WD	Female	17	-0.57	0.87	-0.81	-1.25	0.39	-0.61	-0.78	0.83	0.33	-0.61	0.13
1715WD	WD	Female	30	-0.8	0.38	-1.7	-0.4	-0.01	0.3	0.02	-0.89	-0.8	0.21	0.58
1776WD	WD	Female	39	0.29	-2.43	-0.66	0.29	0.29	0.08	0.34	0.44	-0.51	0.26	-0.12
2452WD-H	WDHEP	Male	43	1.52	0.23	1.2	0.25	-1.67	0.66	1.28	1.56	-0.32	1.52	2
1864WD-H	WDHEP	Male	30	0.76	-0.32	1.4	-0.32	-1.77	0.08	0.89	0.24	-0.25	-0.03	0.14
970WD-H	WDHEP	Male	36	-0.6	-0.55	-0.1	0.58	0.79	-2.59	-0.78	0.63	0.11	0.68	0.54
2210WD-H	WDHEP	Male	44	-1.09	-0.6	0.8	2.33	0.58	-1.59	1.64	0.22	-2.75	0.75	-1.49
2481WD-H	WDHEP	Male	43	-1.1	-1.44	-1.49	-0.52	-0.85	0.1	-0.36	-0.12	0.54	1.3	1.93
1779WD-H	WDHEP	Male	29	1.31	-1.38	-0.8	0.11	-2.51	-0.33	1.98	0.98	0.14	0.54	0.66
2415WD-H	WDHEP	Male	46	0.53	-1.16	-0.21	-1.37	-1.58	0.46	2.66	1.69	1.24	0.77	1.42
1780WD-H	WDHEP	Female	23	0.38	-0.31	-1.73	-0.27	-1.56	0.27	-0.19	0.4	-0.04	0.93	0.95
1866WD-H	WDHEP	Female	24	0.39	-0.63	0.89	-0.38	-1.81	0.43	1.9	1.91	0.86	1.54	1.79
1658WD-H	WDHEP	Female	43	-0.62	-2.17	-0.93	0.94	-1.55	0.22	1.98	1.39	-1.09	0.43	-0.42
1868WD-H	WDHEP	Female	45	1.51	0.34	1	1.16	0.43	0.39	0.02	-0.42	-0.87	-0.45	-0.37

1639WD-H	WDHEP	Female	19	0.68	0.91	1.18	2.31	-1.89	-0.58	1.27	0.78	-2.96	1.36	-1.96
2065WD-H	WDHEP	Female	20	-0.6	0.6	0.54	0.75	-0.39	0.04	0.68	1.13	-0.1	0.76	1.68
2438WD-H	WDHEP	Female	19	-0.24	0.44	-3.36	-0.53	0.29	-0.03	-2.09	-1.05	1.94	0.45	2
2086WD-H	WDHEP	Female	32	-0.39	-0.2	1.27	0.3	0.41	-0.7	0.02	-0.08	-0.48	0.86	0.64
972WD-H	WDHEP	Female	18	0.86	-1.09	-0.2	0.72	-0.01	-2.05	-0.68	-0.8	0.5	1.3	-0.86
1843WD-H	WDHEP	Female	31	0.44	-0.88	-0.31	0.23	-0.6	0.18	-0.11	-0.53	0.23	0.33	-0.17
2051WD-H	WDHEP	Female	56	-0.83	1.3	0.67	1.12	0.38	0.61	-0.05	-1.01	-1.04	0.82	0.05
1982WD-N	WDNEU	Male	34	0.73	-0.16	0.69	1.97	-0.19	-0.51	0.11	-0.26	-2.08	0.63	-0.76
2123WD-N	WDNEU	Male	24	-1.37	0.41	-1.03	-1.05	-0.04	-1.13	-0.41	-0.02	0.85	-0.34	0.32
2245WD-N	WDNEU	Male	23	2.4	-0.59	0.51	1.86	0.52	-0.49	0.85	0.21	-1.28	0.9	0.91
2309WD-N	WDNEU	Male	44	-0.18	-1.29	0.33	-0.28	-0.84	0.23	-0.3	0.99	-0.48	0.7	-1.26
2506WD-N	WDNEU	Male	52	-0.36	-0.19	0.24	-1.67	-0.75	-0.18	0.35	1.04	0.85	0.11	0.7
2534WD-N	WDNEU	Male	21	0.94	-0.02	0.4	-0.92	-0.5	-0.42	-0.49	0.85	1.05	0.3	1
2291WD-N	WDNEU	Male	36	-0.2	0.28	0.21	0.71	-0.1	0.03	-0.48	-0.08	-0.37	0.48	0.06
205WD-N	WDNEU	Male	44	-1.43	0.14	0.73	-1.03	0.48	-1.71	-0.71	0.74	0.98	0.02	0.26
1167WD-N	WDNEU	Male	28	-1.22	-0.63	0.87	1.79	0.12	-1.43	1.01	-0.77	-1.62	0.58	-0.79
1998WD-N	WDNEU	Male	27	-1.17	1.04	0.35	0.52	0.49	-2.79	1.49	0.67	0.13	0.06	-0.82
2024WD-N	WDNEU	Male	52	2.84	1.78	1.26	1.45	-0.2	-0.14	0.06	-0.22	-1.2	0.88	-0.01
1116WD-N	WDNEU	Female	25	1.25	-0.18	1.75	-0.58	-0.4	-2.61	0.42	1.03	0.46	0.13	-0.52
2001WD-N	WDNEU	Female	44	0.2	-1.01	0.12	0.04	-1.17	-0.2	0.66	-0.06	-0.23	-0.24	0.05
2354WD-N	WDNEU	Female	32	0.71	-0.4	-1.46	0.3	0.06	-0.06	-1.67	0.1	-0.03	-0.27	0.07
2243WD-N	WDNEU	Female	46	-0.32	0.56	-0.56	-1.24	-0.12	-0.34	0.17	-2.38	0.9	0.02	-0.32
2301WD-N	WDNEU	Female	33	-0.53	1.4	-1.7	-1.37	-0.48	-0.22	-1.98	0.22	0.73	0.23	0.49
2508WD-N	WDNEU	Female	23	-0.35	-0.32	1.61	-0.23	-0.2	0.15	-0.41	0.62	0.17	-0.09	0.01
1783WD-N	WDNEU	Female	46	-0.38	1.67	1.05	-0.08	-1.79	-0.08	0.57	1.35	-0.36	0.28	-0.23

Glycine	L-Alanine	Succinic acid	L-Lysine	L-Aspartic acid	L-Glutamine	L-Serine	L-Methionine	Ornithine	L-Tryptophan	L-Phenylalanine	L-Tyrosine	Urea	L-Cysteine	b-Alanine	Glycerol
-0.66	-0.62	-0.72	-2.63	-1.77	0.53	-0.19	-0.19	0.37	-0.18	-1.18	-0.97	-0.19	1.35	-0.24	0.19
-1.26	0.42	-0.05	-0.21	-2.19	1.15	-1.35	0.43	-0.13	0.57	-0.97	-0.68	-0.42	2.06	0.84	-1.24
-1.77	0.54	-1.72	-0.29	-1.97	1.05	-0.85	-0.09	-0.09	1.15	-1.33	0.15	-1.41	1.83	-0.9	0.42
-1.19	-1.16	-0.36	0.46	-2.14	0.37	-1.28	-1.7	-0.67	-0.21	-1.09	-0.79	1.28	-0.95	0.24	-0.53
-0.66	0.53	-0.19	1.02	0.03	0.35	0.83	-0.23	0.45	1.03	-0.16	0.08	1.44	1.09	-1.11	-0.98
0.26	-0.65	0.62	-0.42	-0.38	0.75	0.47	-0.37	0.44	0.22	-0.84	-0.69	-0.1	1.26	-0.43	-0.02
0.18	2.06	0.27	-0.03	-0.85	0.65	1.04	-0.02	0.92	-0.62	-0.62	-0.46	-0.75	1.03	-0.29	-1.7
0.93	-0.09	-1.99	0.83	-2.22	0.16	0.46	-0.49	-0.12	-0.68	-1.23	0.09	0.78	0.81	-1.26	-0.61
-2	-2.82	0.62	-0.22	-2.39	-0.2	-2.39	-1.56	-2.21	-0.71	-1.41	-1.22	1.41	-1.3	-1.6	0.04
-1.04	-0.61	0.55	0.38	-1.47	0.73	-1.5	-0.18	-1.1	-0.01	-1.83	-1.63	0.33	1.51	1.34	-0.25
0.98	-0.83	-0.05	1.21	-1.87	1.16	0.02	-0.55	-0.47	-0.05	-2.67	-1.45	1.09	1.09	-0.2	-0.54
-1.01	-0.48	-0.21	-1.32	0.15	0.56	0.67	-0.25	1.29	-1.15	-0.87	-1.64	-0.72	1.73	-0.46	-0.13
-0.56	-0.24	-1.55	0.66	-1.7	0.88	-1.75	-0.37	-1.53	0.24	-1.83	-1.24	0.76	1.45	0.44	-0.24
0.36	-0.09	0.36	0.28	-1.92	0.47	-1.03	-0.44	-0.32	0.87	-1.4	-0.84	-0.48	1.49	-1.1	-0.06
-1.52	-1.06	-2.42	0.46	-1.67	0.67	-0.53	-0.44	-0.24	0.51	-1.84	-0.92	-0.71	1.18	-0.37	0.09
-0.02	1.11	0.56	0.23	0.74	-2.81	1.05	0.51	-0.22	0.01	1.01	1.09	-0.14	-0.7	0.57	0.16
0.69	0.66	0.27	1.87	-0.07	0.27	0.46	0.17	0.2	0.02	0.79	0.26	1.75	-0.67	0.68	-0.35
-0.89	-0.34	-0.33	-0.47	0.21	0.65	-0.43	-1.23	0.03	-0.23	-0.47	-1.53	-1.38	1.53	-0.7	0.32
-0.78	-0.58	0.79	0.4	0.81	-0.78	-0.42	-0.46	-1.18	0.14	0.5	-0.08	1.46	-1.54	0.87	1.36
-0.42	0.55	1.32	1.01	0	-1.87	-0.48	-0.14	0.16	0.63	0.24	-0.5	-0.89	0.29	-1.15	-0.21
0.33	1.13	0.83	0.72	0.05	-0.2	0.85	-0.28	0.31	0.84	0.73	-0.1	-1.47	-0.81	-1.71	-0.02
-0.15	0.47	-0.66	0.01	-0.23	-0.03	0.68	-0.62	0.41	0.33	0.24	-0.19	-0.35	-0.21	0.59	0.1
-0.47	1.08	0.36	-1.92	0.17	0.27	-0.14	-0.28	-0.3	0.85	0.24	-0.2	-0.08	0.45	-0.41	0.42
-0.72	-0.65	-0.29	-2.45	-0.19	0.52	-1	-1.45	-0.64	0.21	0.05	-1.51	1.35	-0.32	0.48	0.1
-0.43	-0.41	1.05	0.29	0.55	-0.58	0.32	-0.24	-0.69	-0.18	0.32	-0.27	0.02	-0.87	1.06	0.98
-0.66	-0.77	0.04	-0.28	0.93	-0.13	-2.15	-0.51	-0.48	-0.42	0.08	-1.47	-0.13	0.06	0.05	-2.78
1.74	-0.83	1.55	-0.16	0.77	-0.07	-0.78	0.98	0.9	-1.58	1.3	0.99	0.14	0.22	-0.71	1.16
1.27	-0.92	-0.09	0.45	0.34	1.11	-0.96	2.68	0.94	-0.72	0.62	1.8	1.37	0.85	2.13	-0.02
-1.27	0.03	0.75	0.61	0.32	-0.6	0.26	-0.34	0.47	1.13	-0.71	-0.47	-1.54	0.59	-0.56	-1.16
-0.8	-0.51	-1.93	1.22	1.11	0.38	-0.29	0.3	0.41	1.38	0.19	0.33	0.92	-0.06	0.91	0.27
2.41	1.45	1.54	-2.08	1.24	-0.22	0.49	-0.41	-0.13	0.18	1.56	0.06	-0.33	0.3	0.17	0.78
0.94	0.5	1.01	-0.61	0.86	0.66	1.61	3.17	1.17	2.18	1.63	2.55	-0.2	-0.26	1.33	-0.43
0.7	1.53	0.58	1.03	0.78	1.16	-0.37	2.82	0.91	-0.23	1.48	1.49	-2.1	0.32	-1.05	0.37
2.58	-0.92	1.09	-0.92	0.39	0.97	0.59	0.25	0.36	1.05	0.88	1.16	0.64	0.26	-0.34	1.43
0.6	1.68	-1.24	0.66	1.13	0.64	3.17	3.62	0.81	1.19	2.8	2.78	-0.23	-1.13	0.11	0.44
0.68	0.02	0.34	-3.39	0.18	-4.31	-1.57	1.65	-5.46	-3.15	-0.14	1.65	-0.11	-2.46	-2.05	0.46
0.95	-0.01	-0.22	-1.35	0.39	-0.17	-0.5	-0.32	0.05	-1.52	-0.16	-0.76	1.75	-1.33	2.19	0.25

-0.12	1.23	-0.05	-0.38	1.07	0.82	1.21	0.9	1.03	1.26	1.5	1.9	-1.24	-0.6	-0.35	0.5
-0.23	-1.26	1.84	-0.34	0.43	0.09	-0.1	0.02	0.87	-3.57	0.22	-0.73	-1.92	-0.29	-0.32	2.07
1.9	2.18	2	0.14	-0.2	-0.31	1.61	-0.47	-0.79	0.31	-0.22	0.03	-0.52	0.84	-0.67	0.53
0.53	0.99	0.94	0.11	0.51	-0.84	0.03	-0.02	0.05	-0.04	0.57	-0.27	-0.31	-0.98	0.97	0.52
0.12	1.41	1.31	0.04	0.39	-1.87	0.98	-0.55	-0.51	-0.33	-0.08	-0.45	-0.89	-0.44	-0.46	0.63
0.88	-0.61	0.4	0.51	0.77	0.22	1.37	-0.01	1.18	-0.04	-0.03	0.29	-1.6	-0.04	-0.78	0.42
-0.12	0.5	0.11	1.04	-0.02	-0.69	-0.18	-0.41	0.54	-0.14	0.12	-0.02	0.95	0.11	1.43	0.54
0.03	-0.99	-1.17	0.93	0.48	-0.06	-0.03	-0.18	0.85	-0.22	0.44	0.46	1.26	-0.87	1.43	-0.36
-0.42	0.43	-0.8	0.4	0.5	-0.18	-0.99	0.28	0.27	-0.01	0.37	0.26	-0.28	-1.01	1.15	0.55
0.41	-1.19	0.62	1.45	1.51	-1.75	0.93	0.23	0.61	0.4	1.74	0.38	1.14	-0.62	-0.95	-0.58
-1.09	-1.29	1.55	-0.1	0.23	-0.28	-0.25	-0.72	-0.61	0.87	0.05	0.56	-0.05	-0.96	-1.57	-0.02
-0.99	0.47	-0.84	1.35	0.44	0.84	0.17	0.74	-0.45	1.77	0.32	1.36	0.2	1.12	-1.08	-0.22
-0.34	-0.6	-0.09	0.04	0.34	0.19	1.19	-0.37	0.99	-0.15	0.16	0.79	-0.94	-0.43	-0.55	1.18
-0.72	-0.08	-0.23	0.81	0.28	0.01	0.16	-0.33	0.4	-0.19	0.22	-0.14	-0.38	-0.74	-0.04	-0.19
0.12	0.36	-1.07	0.58	0.29	0.22	0.79	-0.26	0.64	-0.02	0	-0.39	-0.15	0.15	0.21	-0.66
1.24	-0.11	0.05	-0.53	0.73	-1.27	0.97	-0.74	-0.72	-0.58	-0.03	0.01	1.05	-1.66	0.62	0.82
1.63	1.08	-0.07	0.68	0.6	-1.45	0.73	-0.08	-0.75	-1.07	0.92	-0.39	1.62	-1.42	1.91	0.38
-0.29	0.24	0.63	-0.41	0.58	-0.86	-0.52	-0.22	0.41	-0.01	0.43	0.25	0.89	-0.52	1.05	0.87
-0.8	1.4	-0.42	-1	0.66	0.55	0.28	0.05	-0.15	0.84	0.08	0.69	0.22	-0.13	0.01	-0.78
-0.49	-2.5	-1.73	-0.3	-0.17	0.6	-0.67	-0.94	-0.11	-0.21	-0.33	0.18	1.06	-0.99	1.8	0.02
-0.1	-0.69	-2.06	-0.22	0.15	-0.08	0.14	-0.73	-0.07	-0.95	-0.84	-0.66	-0.21	-0.9	-0.15	0.61
0.14	0.65	-0.28	-0.3	0.51	0.04	-0.71	0.08	0.08	-0.96	-0.14	0.09	-0.42	0.02	1.08	0.15
-0.83	-0.29	-0.32	0.12	0.63	0.24	-0.65	-0.27	1.02	-1.4	0.16	-0.37	-1.85	0.04	-0.75	0.78
0.41	-0.1	-0.57	-0.03	0.24	0.32	-0.39	-0.48	-0.22	0.08	-0.64	-0.39	0.16	0.18	-0.75	-0.88
1.79	-0.41	-0.21	0.37	0.93	1.37	0.91	1.09	0.82	1.26	1.1	1.67	-0.56	0.03	-0.61	-4.95

L-Leucine	L-Histidine	L-Proline	L-Asparagine	Citric acid	L-Valine	Cholesterol	L-Threonine	Arachidonic acid	D-Glucose	Ketoleucine	Taurine	Palmitic acid	Citrulline	Uric acid
0.6	-1.02	0.02	1.51	0.06	0.85	-1.07	0.06	0.59	1.08	0.57	-2.08	-0.19	-0.77	0.21
1.7	0.6	1.15	1.65	-0.88	1.81	-0.82	0.66	0.33	1.39	1.5	0.27	-0.63	-0.07	0.76
1.12	0.16	-0.15	1.51	-1.33	1.39	1.32	0.48	0.69	1.96	-0.95	-2.69	1	-0.78	2.2
-0.55	-0.29	-0.25	0.47	0.19	2.02	-0.34	0.12	0.03	0.83	1.06	1.03	0.78	2.45	0.89
2.07	0.93	1.69	1.66	-1.8	2.27	-0.44	0.34	-1.36	0.67	0.18	1.11	-1.56	-0.71	0.31
0.02	0.23	-0.09	-0.08	-0.21	0.36	-1.5	-0.44	-0.35	1.35	0.42	-0.2	-0.43	-1.12	0.85
0.35	-0.39	2.08	2.09	0.88	0.73	-0.27	1.55	-1.31	0.46	-1.75	-0.32	-3.03	-1.08	-0.29
0.44	-0.41	-0.52	-0.16	-0.31	-1.01	-0.68	-0.29	-1.82	0.82	0.31	0.45	-0.74	-0.09	-1.18
-0.63	-1.65	-0.77	-0.75	-0.41	-0.89	-0.56	-4.01	1.63	1.46	1.39	0.59	0.8	-0.55	0.62
-0.73	-0.96	-0.14	1.25	0.29	-0.71	1.24	0.42	-0.64	0.86	0.12	-0.74	0.56	-0.35	0.17
-0.67	-0.06	-0.33	0.77	0.17	0	-0.44	-0.85	-0.09	1.27	0.89	0.42	-0.31	-0.01	0.64
0.51	-0.24	-1	1.29	-1.03	0.3	-0.36	0.79	0.26	-1.09	0.6	0.66	0.21	0.47	0.37
0.08	0.56	-1.16	0.66	-0.33	0.98	-0.87	-0.4	0.62	0	1.59	0.4	0.19	-0.79	0.12
-0.29	0.63	-0.67	0.19	-0.73	0.44	-1.33	1.27	-0.33	1.85	1.39	0.44	-0.31	-0.47	1.09
-0.13	-0.55	-0.8	0.05	0.41	0.68	-0.03	0.47	0.15	0.67	1.05	-0.26	0.43	-0.76	0.92
0.86	-0.8	-0.39	-1.43	0.98	-0.25	-1.86	0.22	0.31	0.06	1.22	0.72	0.15	2.73	-0.82
2.5	0.01	0.86	-0.27	-0.72	1.91	-2.67	0.35	-1.14	-0.76	0.84	0.78	-0.43	0.77	-0.28
-0.82	-0.71	-0.9	-1.41	-0.25	-0.11	0.84	-1.32	-1.88	0.38	1.01	0.82	0.42	-0.86	1.54
-0.45	-0.27	-0.13	-0.5	-1.64	-0.42	-0.19	-0.32	1.03	-0.42	-1.13	1.1	0.73	2.65	-0.06
0.03	0.68	0.24	0.27	-0.86	0.76	0.85	0.77	-0.15	0.61	0.72	1.11	-0.23	-1.07	2.15
1	0.18	0.65	-0.34	-0.03	1.32	-0.77	-0.11	0.89	-0.37	0.61	0.54	-1.11	0.52	1.6
-0.41	-0.4	-0.32	-0.13	-1.26	-0.97	0.3	-2.13	-1.73	-1.03	0.4	0.54	-0.19	0.23	1.13
0.3	1.2	0.5	0.77	-1.13	0.6	0.11	-0.16	1.2	-0.51	0.28	-0.35	-0.26	-0.02	1.61
-0.86	0.52	-1	-0.61	-0.15	-0.63	0.31	-2.89	0.11	0.73	0.48	0.18	0.47	0.23	0.6
-0.2	0.23	-0.43	-0.72	-0.23	0.45	-0.02	-0.39	-0.04	-0.01	-0.07	0.4	0.88	0.08	0.26
-0.58	0.2	-1.66	-0.37	1.02	-1.11	1.56	-0.23	1.38	1.23	0.6	0.36	0.94	-0.87	0.07
-0.88	1.39	0.33	0.43	1.72	-1.93	2.11	0.04	2.26	-0.28	-0.8	0.86	0.87	-0.35	-1.31
-1.58	-0.04	0.14	0.35	0.83	-1.03	-1.36	0.42	0.46	-1.89	-1.49	-0.42	0.48	1.93	-2.01
0.62	0.84	-1.37	-0.52	-1.32	0.34	1.36	0.33	0.99	0.83	0.77	0.58	-0.56	-0.33	1.53
1.88	0	-0.04	0.14	-0.64	1.49	-0.33	0.16	0.04	-0.78	-0.37	0.29	0.32	1.69	0.36
-1.31	1.09	0.17	-0.53	0.33	0.19	1.15	0.29	0.29	-0.86	-1.47	0.57	-0.43	-1.13	-0.09
-0.88	1.68	1.83	0.74	1.92	-1.21	0.45	0.86	-0.41	0.05	-0.34	-2	-2.75	0.01	0.64
0.07	1.9	2.65	2.19	1.16	-1.11	0.3	1.46	0.74	-0.47	-1.26	-0.05	0.11	0.53	-1.25
-1.73	1.37	1.15	0.69	0.63	-0.8	0.94	0.47	-0.39	0.49	-0.52	0.61	0.33	-1.15	-0.03
0.71	1.84	2.96	1.86	1.47	0.64	-0.43	1.77	-0.56	0.99	-0.37	0.09	-1.9	0.19	-1.11
-0.82	-3.19	-0.32	-2.69	1.73	-1.16	-0.47	0.6	0.83	-0.13	-1.37	-2.97	1.36	0.98	-2.19
-0.37	0.52	0.29	-1.81	0.01	-0.65	-1.31	-1.12	-0.06	-1.54	-0.87	1.05	0.19	-0.51	-0.33

-0.48	0.14	1.87	0.6	0.85	-0.46	0.38	1.29	-0.06	-0.08	-2.97	-0.53	-0.02	1.09	-0.86
-1.42	-0.58	-0.84	0.36	0.82	-1.85	1.67	-0.19	1.87	-2.38	-0.63	-1.28	2.66	-0.94	-1.17
-0.11	0.66	-1.34	-0.5	-0.35	-0.17	1.91	-0.28	-1.87	-0.13	1.95	0.95	-0.38	-1.32	1.39
0.77	-1.55	-0.07	-1.62	-1.54	0.84	-0.51	0.44	0.5	-0.74	-0.75	0.95	-0.44	0.78	0.85
-1.38	0.38	0.19	-1.51	-1.15	-0.73	-0.08	-0.52	-0.62	0.16	-0.85	0.58	0.3	-0.02	-0.17
-0.05	0.97	-0.99	-0.27	0.38	-0.43	1.09	0.86	0.4	-1.74	-0.34	0.21	-0.95	-1.71	-0.96
0.89	0.34	-0.63	0.09	-0.76	0.36	-0.22	0.05	-0.26	-0.84	0.36	0.49	0.17	1.8	0.17
0.66	0.29	0.02	-0.22	0.14	0.06	-0.7	-0.69	-0.83	-1.32	0.08	0.36	0.97	0.92	0.13
0.38	-0.69	-0.78	0.1	0.37	0.21	-0.74	0	-0.09	1.03	0.14	0.23	0.2	1.26	-0.9
2.43	0.49	-0.61	0.35	-2.83	2.3	-0.21	0.05	-0.98	-1.12	0.19	1.09	-0.26	-0.55	-0.5
-0.51	0.97	-1.75	-0.82	0.63	-0.27	1.08	-0.61	-2.87	0.35	0.62	-0.05	0.45	-0.69	0.68
0.72	1.54	1.74	-1.5	-0.52	0.59	0.87	1.85	-0.58	0.36	-1.71	-0.26	-1.76	-0.26	-0.46
0.47	-0.52	0.48	0.12	-0.05	-0.26	0.77	0.08	0.21	1.42	1.2	-0.62	1.89	-0.86	0.31
0.49	-0.81	-0.79	0.4	-0.55	-0.62	-0.22	-0.72	0.07	-0.37	1.16	-0.7	0.57	0.33	-0.09
0.52	-0.55	-0.14	-0.96	-1.5	0.4	-0.33	-0.67	-1.35	0.86	0.15	-3.3	-0.09	-0.45	-1.09
0.23	-2.12	0.07	-1.17	-0.91	-0.25	-1.94	0.39	-0.82	-0.52	-0.1	0.92	1.04	0.69	-1.45
0.96	-1.33	0.24	-0.39	1.14	0.1	-0.58	-0.56	-0.15	-1.22	0.05	0.75	-0.24	-0.28	-0.39
0.65	0.1	0.24	0.14	0.41	-0.11	-0.3	-0.56	1.11	-2.11	-0.98	-0.24	-0.84	-0.94	0.12
-0.01	-1.44	-0.02	-0.91	1.3	0.14	-0.83	-0.51	1.05	1.08	0.49	-0.09	-0.97	0.29	-0.26
-2.53	-0.66	0.06	-0.02	0.96	-2.24	-0.14	-1.03	0.66	-0.55	0.06	-0.56	1.58	0.54	-1.79
-0.84	-0.14	-0.73	-0.48	1.13	-1.02	1.04	-0.34	1.19	-0.81	-0.24	0.33	1.51	-0.3	0.39
0.09	-1.67	-0.66	-0.37	0.63	-0.12	0.66	1.33	-0.3	-0.52	-0.42	-1.68	0.11	-0.26	-1.49
-0.78	-1.02	-0.89	-0.07	0.96	-0.71	0.15	0.35	1.51	-0.61	-0.76	-0.76	0.26	0.88	-0.84
-1.63	0.29	-0.17	-0.76	1.06	-0.73	1.52	-0.44	0.42	0.27	0.25	0.16	-1.11	-1.44	-0.39
-0.5	1.17	1.22	1.18	0.84	-0.57	0.91	1.19	-0.76	-0.38	-2.19	-0.82	-0.84	-0.19	-0.95

L-Isoleucine	N-Acetylornithine	Isocitric acid	N-Acetylglutamic acid	Malate	Oleic acid	Creatinine	Indoleacetic acid	g-Hydroxybutyric Acid	3-Hydroxybutyric acid	4-Hydroxyproline	Cystine
0.52	-0.63	-0.23	1.57	0.03	0.5	-0.28	0.02	0.63	0.92	-1.4	-0.35
2.19	-1.27	-1.2	0.14	-0.16	-0.8	0.21	0.2	-1.06	-1.11	0.77	0.16
1.19	0	-1.95	1.59	-1.08	1.34	0.57	0.04	-0.74	0.18	-1.05	1.83
0.36	-0.23	0.13	-1.04	-0.32	-1.74	-0.64	0.53	1.13	-0.14	0.75	0.18
1.35	-1.9	-1.16	-2.63	-0.38	-0.41	0.23	0.1	-1.47	-0.83	-0.45	-0.46
0.23	-0.47	-0.44	0.36	0.41	0.36	0.27	0.66	-1.72	-1.74	-0.76	0
0.69	-0.74	0.35	0.32	-0.71	-2.52	-0.04	0.33	-0.82	-0.08	1.12	-0.13
-2.83	-1.76	0.76	1.12	0.08	-1.16	0.42	1.46	-0.34	-0.34	0.03	-0.37
-0.72	-2.52	-3.29	1.38	0.49	1.3	0.35	1.74	-0.05	2.65	0.49	-0.65
-0.49	0.69	-0.38	0.62	-0.64	-0.07	-0.19	0.31	1.19	-0.28	-0.07	0.02
0.09	0.03	0.01	0.64	-0.36	-0.34	0.33	0.23	0.52	0.31	-1.05	0.17
0.59	1.2	-1.51	0.59	0.47	0.16	0.47	0.5	0.33	-1.14	-0.02	0.1
0.17	-1.82	-0.59	0.16	-0.16	0.88	0.84	0.42	-1.77	1.23	-0.94	0.15
-0.42	-0.08	-1.43	1.57	-0.03	0.54	-0.05	0.93	0.43	-1.18	0.08	1.54
-0.1	0.61	-0.67	0.16	-0.56	0.85	-0.08	1.32	0.49	1.63	-0.46	1.07
0.74	-1.08	1.19	-0.68	0.4	0.23	-1	-1.22	0.85	-0.37	2.3	-1.59
2.34	0.54	-0.58	0.54	-0.1	-2.1	-1.28	1.03	0.49	-0.72	-0.19	-1.63
-1.36	-0.3	0.58	0.13	0.76	1.17	-0.27	-1.8	-0.5	2.55	-1.07	-0.54
0.31	-0.38	-1.11	0.08	-2.46	0.04	-0.33	1.44	1.18	-1.29	-0.9	-1.22
-1.21	0.14	-0.39	-1.12	-0.27	0.63	0.26	2	-0.06	-0.77	1.72	0.8
0.31	0.07	0.1	0.11	-0.5	-0.13	0.86	0.61	-0.49	-0.29	0.2	-0.28
-1.29	0.59	-0.67	-0.19	2.8	-0.4	-0.26	-0.53	0.63	-0.11	-0.64	-0.45
-0.15	-0.84	-0.43	-0.63	-3.08	0.33	1.65	-0.05	-0.49	-0.28	0.57	0.8
-0.74	-1.84	0.16	-0.51	-0.98	-0.44	0.85	-0.26	0.31	0.06	-0.51	0.73
-0.32	0.33	0.01	-1.19	-0.49	1.31	0.41	-0.43	0.85	0.86	-2.15	-1.25
-0.84	0.3	1.09	-0.47	-0.38	1.11	0.44	-0.98	0.51	0.52	-0.05	0.37
-0.87	-0.2	1.93	1.91	0.78	1.78	-0.7	-0.1	-1.72	-1.11	1.79	2.72
0.74	0.8	0.34	0.44	-0.29	-0.27	-1.44	-0.14	1.15	0.08	0.64	0.36
0.72	1.01	-0.72	-0.43	0.48	-0.68	0.67	3.25	-0.01	-1.15	-0.63	0.71
1.54	0.35	-0.43	-1.1	-1.13	-0.91	0.85	1.45	0.46	0.09	-0.72	-0.13
-2.32	0.2	0.16	-4.66	-0.18	0.65	0.52	-0.79	-1.8	-1.59	-0.41	-0.12
-0.35	-0.63	2.17	-0.02	1.12	-2.35	0.86	1.14	-0.55	-0.54	1.83	0.24
-0.46	-0.32	0.97	-0.22	0.73	0.75	0.5	-1.54	-1.58	0.11	1.68	3.03
-1.16	-0.96	0.51	-0.05	0.53	0.55	0.06	-0.45	-2.24	0.65	0.85	0.19
0.75	-0.78	1.46	-0.98	0.69	-1.9	-0.45	0.8	-1.46	-0.19	1.19	0.81
0.64	-0.06	1.39	0.98	1.2	1.17	-4.88	-2.03	0.66	-0.82	0.7	0.2
0.49	0.93	0.61	0.18	-0.08	-0.76	0.32	-0.56	0.14	-0.25	-1.38	-1.38

-0.51	0.55	-0.05	-0.7	-0.14	-0.48	-0.42	0.11	-0.11	-1.11	0.67	0
-1.22	-0.53	0.94	0.15	1.9	2.12	-0.66	-1.81	0.42	2.4	1.75	0.96
-0.7	-1.81	0.43	0.12	1.46	0.24	1.06	0.13	-0.83	-1.1	-0.55	-0.58
0.48	1.91	-0.87	0.21	-0.55	-0.51	-0.23	0.31	1.19	-0.29	-1	-0.92
-0.64	0.61	-0.65	-0.79	0.11	0.55	-3.3	0.05	-0.9	1.23	-0.85	-1.11
-0.9	0.79	0.24	0.12	1.52	0.16	0.46	-0.95	-0.81	-0.4	0.16	-0.46
0.78	1.82	-0.66	-0.13	-0.24	-0.74	0.44	-2.11	1.36	-0.46	-1.26	-0.85
0.94	0.63	0.4	-0.18	-0.39	-0.9	-0.22	-0.41	0.79	0.05	-0.57	-0.99
1.27	0.52	1.07	0.35	-1.69	-0.43	0.63	-0.89	1.26	0.24	0.81	-0.88
1.67	1.22	-1.45	-0.57	0.37	0.56	1.44	-0.39	0.96	1.36	-2.6	-0.6
-1.07	0.11	0.8	-1.35	2.41	0.26	0.7	-0.01	0.11	-0.89	-0.64	0.66
0.35	-1.24	-0.15	0.24	-0.11	-1.4	-0.1	0.52	0.07	-0.66	1.04	1.53
-0.08	0.63	-0.62	0.6	0.38	1.94	-1.19	-0.14	0.75	1.09	0.13	0.19
0.09	1.08	-0.01	0.31	-0.47	0.29	0.4	-0.39	0.64	0.91	0.02	-0.69
0.41	0.75	-1.4	0.76	-0.88	0.2	-0.16	0.66	0.65	-0.94	0.15	0.59
0.72	-0.04	0.11	1.02	-0.66	0.3	-1.53	-0.79	0.47	1.13	0.89	-1.82
1.25	-0.62	0.94	1.02	-0.03	-1.09	0.57	-0.58	-1.21	-0.56	0.19	-0.74
0.55	1.32	0.69	-0.7	0.16	-0.61	-0.02	-1.37	1.3	-0.37	0.51	-0.68
0.21	0.61	1.47	0.15	0.7	-0.55	-0.06	0.13	0.16	-0.36	0.89	0.71
-1.11	0.36	0.24	0.19	-0.14	0.2	-0.05	-1.15	1.77	1.63	0.15	-0.85
-1.13	0.94	0.93	0.05	1.29	0.93	1.02	0.38	-0.15	1.41	-0.81	-0.59
0.33	0.39	0.65	0.5	0.61	-0.26	0.18	-0.06	1.43	-0.02	-0.73	-1.45
-0.42	2.4	0.37	-0.22	-1.99	0.96	0.65	-0.86	0.33	0.82	-0.99	0.36
-0.99	-0.25	1.12	0.22	-0.01	-0.27	0.34	0.03	-0.67	-0.02	0.11	0.61
-0.59	-1.12	-1.32	-0.03	-0.27	-0.13	0.04	-0.04	-2.1	-0.61	0.68	1.95

Lactic acid	Stearic acid	Capric acid	Linoleic acid	Pelargonic acid	Threonic acid	Pyroglutamic acid	1-Acylglycerol	Indolelactic acid	g-Tocopherol	Dodecanoic acid	N-Methylalanine
0.15	0.39	-0.08	0.11	-0.1	1.42	1.91	-0.35	0.51	0.2	1.11	0.08
-0.76	0.68	-0.39	-0.15	-0.26	1.02	1	-0.64	1.58	0.11	-0.12	0.7
0.19	0.03	-3.51	1.21	-0.42	0.61	0.96	-0.97	1.34	0.37	-0.85	-1.86
-0.7	1.49	0.12	-0.9	-0.27	0.69	1.43	-0.13	0.38	0.33	0.56	-0.43
0.89	-0.94	-0.15	0.31	-1.45	0.86	1.2	-0.04	0.5	0.56	0.69	0.1
0.87	-0.52	-0.28	0.71	-0.65	1.19	0.85	-0.61	1.46	0.71	-0.13	0.26
0.43	-2.04	0.01	-1.9	0.28	1.1	1.31	-0.71	-0.35	1.83	-1.13	0.63
-0.61	-0.23	2.71	-0.7	-3.64	1.09	1.13	0.6	0.01	0.66	0.85	-1.34
0.32	1.09	0.6	1.11	0.24	1.59	0.78	2.28	1.63	2.06	0.43	0.46
-1.29	1.57	0.37	-1.34	0.86	1.2	0.48	0.37	0.19	-2.27	0.09	0.34
-0.95	0.54	0.21	-3.09	0.49	1.24	0.36	-1.86	0.06	1.01	0.32	-0.33
1.65	0.78	-0.3	0.3	-0.21	1.07	0.91	-0.23	-0.54	0.11	0.17	1.4
-0.55	-0.06	0.21	1.02	-1.3	1.32	-0.4	-1.65	0.56	0.3	0.04	0.73
0.52	-0.44	0.09	0.62	-2.36	2.11	1.48	-1.45	0.81	-0.8	0.2	0.64
-0.05	0.51	-1.95	0.49	-0.39	1.68	-0.24	-2.1	0.55	1.76	-0.19	2.19
0.19	0.67	0.86	0.23	1.25	-1.07	0.49	1.75	-0.74	0.12	0.23	-3.23
-1.01	0.32	-0.3	-1.43	0.07	-0.48	-0.29	1.79	0.1	-1.13	-0.29	0.26
3.31	-0.33	0.89	1.01	1.67	-0.61	-0.06	0	0.72	0.67	0.38	-0.75
-0.1	1.12	0.09	-0.28	0.75	0.47	-0.73	0.18	0.29	0.53	0.17	-0.26
-0.35	-1.11	-1.33	0.34	-0.56	-1.85	-2.17	0.23	1.85	-0.26	-0.26	1.83
0.27	-1.2	-0.56	0.47	-0.68	-0.4	0	-0.9	0.11	1.08	-0.6	-0.08
0.82	0.48	-0.61	-0.35	0.3	0.14	-0.89	2.26	-0.29	-1.24	-1.03	-1.15
-0.81	-0.61	-0.29	0.72	0.31	-0.21	-0.63	0.59	-0.42	0.3	-1.5	1.36
-1.49	0.52	-0.09	0.39	-0.72	0.36	-1.25	0.06	0.26	1.82	-3	-0.99
-2.17	0.08	0.36	1.03	0.48	0.19	-2.27	-0.33	-0.94	-0.45	0.16	0.44
-0.29	-0.03	0.87	0.23	0.64	-2.23	-0.87	-0.98	0.35	0.9	1.4	-0.84
-0.23	-1.03	0.81	1.22	1.94	-1.94	-1.26	-0.04	-2.02	1.77	1.42	-0.38
-1.25	0.5	0.18	-0.51	-0.64	-1.2	0.75	0.62	-0.44	0.76	0.16	1.44
1.34	-0.33	0.85	-0.22	0.67	-0.13	-0.21	0.45	0.25	-0.37	-0.57	-0.06
-1.08	0.84	-0.12	-0.9	-0.43	-0.12	-1.1	-0.48	-0.13	0.35	-0.73	-0.09
0.12	-1.42	-0.43	0.71	-0.44	0.47	-1.83	-0.04	0.19	-0.56	0.47	0.5
0.2	-2.49	-0.74	0.25	-0.37	0.37	0.12	-0.5	3.17	-1.41	-3.42	-1.12
-0.32	-1.36	0.62	0.96	0.77	-0.32	0.17	-1.6	-1.05	0.19	0.81	0.21
-0.67	-0.7	2.34	0.56	-1.13	0.16	0.03	-0.13	0.67	-0.53	1.79	0.31
0.36	-1.41	-1.25	-2.09	0.37	-2.5	-0.29	0.1	0.58	-1.27	-0.42	0.22
-0.42	1.04	-1.99	0.98	1.2	-1.66	1.4	0.54	-2.77	-2.2	1.17	0.28
0.45	0.85	0.08	-0.45	0.71	-0.86	-0.81	0.24	-0.61	-1.06	-0.16	-0.09

-1.63	-0.09	0.03	-0.49	-0.1	-0.99	0.62	0.03	0.32	0.16	0.44	0.43
1.26	0.74	1.65	1.79	1.5	-0.65	0.9	-0.16	-2.81	-0.37	2.64	-0.01
2.45	-1.43	0.24	0.64	1.42	0.63	-0.36	0.23	-0.55	0.1	0.15	0.81
-0.38	-0.27	-0.42	-0.25	-0.6	0.67	-0.15	1.89	-0.1	0.83	-1.04	0.54
2.24	0.36	-0.84	0.85	-1.11	0.23	-2.25	-0.72	-0.3	-1.33	0.84	0.61
1.15	-1.7	0.23	-0.87	-0.42	-0.98	0.19	0.36	0.59	0.5	0.33	-0.02
-0.02	0.93	-0.31	-0.2	0.5	0.74	-0.46	-0.25	0.14	0.5	-1.27	1
-0.41	1.49	0.42	-0.55	0.91	0.34	-0.57	1.43	-0.56	-2.05	-0.09	0.06
-0.29	0.97	-0.81	-1.2	0.66	-0.36	-0.51	-0.03	-0.44	-0.32	-0.29	-3.34
0.82	-0.5	-0.65	0.62	-1.61	-0.52	-1.62	-0.72	0.49	-1.33	-0.52	-0.46
-1.29	0.44	0.08	0.79	-0.13	0.43	-0.85	-1.95	0.73	-0.62	0.8	-1.09
-1.53	-1.68	1.8	-0.74	-0.27	-1.72	-0.45	0.38	0.42	0.93	0.36	-0.05
0.22	-0.05	1.58	1.63	1.2	-0.08	0.4	-0.22	0.03	-0.36	1.4	-0.37
0.7	0.48	0.21	0.76	0.63	-0.06	-0.29	0.12	-0.51	-1.68	-0.77	0.01
0.03	0.73	-1.73	-0.24	-0.5	-0.41	0.7	-0.45	-0.04	0.02	-0.03	0.52
0.11	1.36	0.47	0.12	0.96	-0.72	0.51	2.24	-0.9	-0.71	0.75	0.94
1.04	-0.04	0.33	-1.62	1.28	0.16	2.42	1.26	-0.95	-1.08	-0.21	-0.24
0.44	-0.57	-0.29	0.11	-0.48	0.12	-1.31	1.38	-0.12	0.41	-0.71	-0.21
-0.25	-0.28	-0.53	-0.18	-0.77	-1.2	0.34	0.26	1.06	-0.1	-1.88	0.86
-0.32	2.34	0.59	0.33	-0.4	-0.31	0.18	0.89	-1.2	-1.08	-0.63	0.56
0.67	1.36	0.82	1.12	1.12	0.32	-0.11	-0.15	-1.17	0.37	0.75	0.06
-0.44	0.51	-0.25	-2.32	0.03	0.2	0.27	0.08	-1.24	0.55	0.31	-2.21
-0.75	-0.37	-1.24	1.04	-1.6	-0.87	-0.14	-0.85	-0.95	-0.07	-0.12	-0.11
0.05	-1.09	-0.15	-1.12	0.17	0.14	0.22	-0.34	-0.43	0.87	-0.14	-0.29
-0.84	-0.93	0.89	-0.69	0.63	0.13	0.81	-1.03	0.69	0.91	0.72	0.64

Glycerolphosphate	3-Aminoisobutanoic acid	5-Methoxytryptamine	2-Hydroxybutyric acid	Myristic acid	Arachidic acid	Palmitoleic acid	Pentadecanoic acid
0.44	-0.1	-0.92	-1.05	0.41	1.06	0.46	1.01
-0.18	0.15	-1.31	-1.26	-0.45	1.28	-1.12	0.76
-1.18	0.42	1	-1.86	0.29	-0.14	0.7	1.36
0.43	0	-1.44	-0.47	0.34	0.09	0.02	0.67
1.87	-0.53	-1.47	-1.7	-0.14	-0.97	-0.66	0.44
0.95	-0.8	-1.04	-1.08	0.57	-0.85	0.24	1.02
1.19	0.03	0.75	-1.19	-1.71	-1.43	-2.16	0.25
0.25	-0.4	-1.15	-0.99	0.48	-0.16	-1.64	0.45
0.28	-1.41	-1.13	1.23	0.62	0.07	0.11	1.01
0.82	1.52	0.85	-1.81	0.28	0.82	-0.04	-0.74
0.56	-0.9	0.36	-0.83	-0.28	0.28	-0.95	0.92
1.62	-1.17	-0.14	-0.11	-0.81	0.62	-0.55	-3.71
0.93	0.26	0.75	1.28	0.68	-0.02	0.36	0.41
1.15	-1.49	1.5	-0.81	0.16	-1.39	0.78	1.29
1.25	-0.03	1.21	0.2	0.87	0.16	0.69	0.87
-1.79	-0.67	-1.4	0.56	0.08	0.59	0.41	0.14
-0.88	-0.47	-0.86	-1.63	-1.19	0.52	-1.39	-0.3
0.27	0.21	0.76	-0.3	0.05	-0.34	0.27	0.45
-0.68	-0.82	-1.77	-0.48	0.22	0.38	0.4	0.23
0.56	0.53	0.27	0.9	-0.43	-2.39	0.13	0.21
-3.67	-3.19	1.1	-0.47	-0.61	-1.32	-0.41	-0.45
1.2	-1.17	1.49	0.03	-0.42	0.62	-0.02	-0.09
0.63	1.72	0.97	1.38	-0.08	-0.26	0.61	-0.05
0.75	-0.37	1.92	0.25	-0.56	0.3	-1.81	0.44
-0.75	1.02	-0.05	1.54	0.61	-0.58	1.05	0.16
0.51	0.61	0.94	0.16	0.81	-0.82	1.17	1.04
-1.19	0.21	0.35	1.26	1.72	0	1.76	0.74
-0.09	0.32	0.13	0.34	0.08	0.95	0.25	-0.77
0.95	-2.39	0.72	-0.24	-0.46	-0.53	-1.01	0.35
0.84	0.33	0.38	-0.24	-0.7	0.68	-0.99	-0.41
-1.04	-0.07	0.61	0.23	0.09	-1.12	0.73	0.21
-0.76	-0.04	-0.18	-1.18	-1.83	-2.51	-1.53	-0.12
-1.42	1.43	-0.15	0.2	0.73	-1.24	1.44	0
-0.02	-0.09	0.22	0.51	1.73	-1.34	0.63	1.14
-0.42	1.22	0.9	0.04	-1.08	-0.93	-1.13	0.43
-1.67	2.73	-1.88	1.51	1.24	0.85	1.29	0.46
-0.85	0.1	-1.01	0.29	-0.48	0.94	-0.37	-0.81

0.04	0.19	-0.25	-1.86	0.17	0.71	0.39	0.06
0.56	0.52	0.17	1.17	2.73	0.44	2.28	0.01
1.2	-0.37	1.28	1.62	0.22	-0.87	0.86	-0.06
-0.93	1.49	-1.12	-0.27	-0.48	-0.82	0.3	-0.37
0.7	0.5	0.22	0.57	0.43	0.55	0.45	0.53
-0.09	-0.64	0.26	-0.11	0.1	-1.03	0.69	-0.79
-0.96	0.45	-1.38	0.4	-0.93	0.74	-0.15	-0.3
-0.6	0.42	-1.95	0.54	0.1	1.9	-0.39	-0.34
-0.54	0.04	-0.54	0.02	-0.81	0.79	-2.09	0.41
-1.43	1.29	-0.87	1.45	-0.31	-0.47	-0.22	-0.57
0.98	-0.82	0.82	0.37	0.21	0.67	0.36	0.15
0.01	1.25	0.63	-1.2	0.15	-0.98	-1.54	-2.31
0.07	-1.83	0.8	0.2	1.86	-0.56	1.88	-2.78
-0.74	-0.69	-0.05	1.98	-4.65	0.41	0.67	0.1
1.49	-0.53	1.15	-0.68	0.08	1.15	-0.16	-3.69
-0.31	0.98	-0.75	-0.29	0.58	1.91	0.39	0.49
-1.04	0.81	-1.33	-2.19	-0.39	1.52	-2.54	-0.44
-0.08	-0.41	0.26	-0.17	-0.65	-0.07	-0.11	-1.15
0.04	-0.37	1.79	1.17	-0.66	-0.37	-0.66	0.67
-0.44	-1.11	-0.59	1.46	0.31	2.66	0.05	0.48
1.08	0.54	0.33	0.36	1.11	0.37	0.77	0.27
-0.25	0.7	-1.07	0.07	0.14	0.87	-0.01	0.23
-0.09	0.34	-0.15	-0.05	-0.11	-0.27	0.74	0.15
0.84	-0.06	1.25	0.35	-0.22	-0.76	-0.17	0.18
-0.34	0.62	-0.21	0.85	0.2	-0.33	0.52	0.06

Supplementary Table S2. Results from SFAM algorithm

Subject ID	Diagnosis	Predicted	Sex	Age	BMI	mtDNA CN	mtDNA deletions	L/P	BCAA/AAA	CP	Total BR	CysCys/Total Cys	Pyruvic acid	L-Glutamic acid
K10No_WD	Control	Control	2	52	-0.51	0.71	1.09	-0.19	1.42	1.45	-0.2	-1.39	0.92	-0.33
K6No_WD	Control	Control	2	38	-1.62	0.98	-0.58	-1.48	1.41	1.06	-1.08	-1.09	1	-1.84
M2No_WD	Control	Control	1	45	1.53	0.79	0.06	0.12	1.27	1.17	-1.42	-1.27	0.46	-0.08
K1No_WD	Control	Control	2	38	-1.16	0.72	-0.34	-0.38	0.96	0.69	-0.62	0.79	0.59	-2.02
K4No_WD	Control	Control	2	31	-0.39	0.26	-1.02	-0.84	1.13	0.89	-0.32	0.45	0.69	-1.86
2275WD	WD	WDNEU	2	17	-0.57	0.87	-0.81	-1.25	0.39	-0.61	-0.78	0.83	0.33	-0.61
2251WD	WD	WDNEU	1	21	1.09	0.47	1.18	0.51	0.35	-0.86	-0.88	0.48	-0.38	0.81
1715WD	WD	WDNEU	2	30	-0.8	0.38	-1.7	-0.4	-0.01	0.3	0.02	-0.89	-0.8	0.21
1776WD	WD	WDNEU	2	39	0.29	-2.43	-0.66	0.29	0.29	0.08	0.34	0.44	-0.51	0.26
1779WD-H	WDHEP	WDHEP	1	29	1.31	-1.38	-0.8	0.11	-2.51	-0.33	1.98	0.98	0.14	0.54
2086WD-H	WDHEP	WD	2	32	-0.39	-0.2	1.27	0.3	0.41	-0.7	0.02	-0.08	-0.48	0.86
2415WD-H	WDHEP	WDHEP	1	46	0.53	-1.16	-0.21	-1.37	-1.58	0.46	2.66	1.69	1.24	0.77
972WD-H	WDHEP	WD	2	18	0.86	-1.09	-0.2	0.72	-0.01	-2.05	-0.68	-0.8	0.5	1.3
1843WD-H	WDHEP	WDNEU	2	31	0.44	-0.88	-0.31	0.23	-0.6	0.18	-0.11	-0.53	0.23	0.33
2051WD-H	WDHEP	WDNEU	2	56	-0.83	1.3	0.67	1.12	0.38	0.61	-0.05	-1.01	-1.04	0.82
205WD-N	WDNEU	WDNEU	1	44	-1.43	0.14	0.73	-1.03	0.48	-1.71	-0.71	0.74	0.98	0.02
1167WD-N	WDNEU	WDNEU	1	28	-1.22	-0.63	0.87	1.79	0.12	-1.43	1.01	-0.77	-1.62	0.58
1998WD-N	WDNEU	WDNEU	1	27	-1.17	1.04	0.35	0.52	0.49	-2.79	1.49	0.67	0.13	0.06
2024WD-N	WDNEU	WDNEU	1	52	2.84	1.78	1.26	1.45	-0.2	-0.14	0.06	-0.22	-1.2	0.88
2508WD-N	WDNEU	WDNEU	2	23	-0.35	-0.32	1.61	-0.23	-0.2	0.15	-0.41	0.62	0.17	-0.09
1783WD-N	WDNEU	WDHEP	2	46	-0.38	1.67	1.05	-0.08	-1.79	-0.08	0.57	1.35	-0.36	0.28

Oxoglutaric acid	Glycine	L-Alanine	Succinic acid	L-Lysine	L-Aspartic acid	L-Glutamine	L-Serine	L-Methionine	Ornithine	L-Tryptophan	L-Phenylalanine	L-Tyrosine	Urea
-0.77	-1.01	-0.48	-0.21	-1.32	0.15	0.56	0.67	-0.25	1.29	-1.15	-0.87	-1.64	-0.72
-0.43	-0.56	-0.24	-1.55	0.66	-1.7	0.88	-1.75	-0.37	-1.53	0.24	-1.83	-1.24	0.76
-0.33	-0.66	0.53	-0.19	1.02	0.03	0.35	0.83	-0.23	0.45	1.03	-0.16	0.08	1.44
-0.72	0.36	-0.09	0.36	0.28	-1.92	0.47	-1.03	-0.44	-0.32	0.87	-1.4	-0.84	-0.48
-0.74	-1.52	-1.06	-2.42	0.46	-1.67	0.67	-0.53	-0.44	-0.24	0.51	-1.84	-0.92	-0.71
0.13	-0.72	-0.65	-0.29	-2.45	-0.19	0.52	-1	-1.45	-0.64	0.21	0.05	-1.51	1.35
-1.02	0.33	1.13	0.83	0.72	0.05	-0.2	0.85	-0.28	0.31	0.84	0.73	-0.1	-1.47
0.58	-0.43	-0.41	1.05	0.29	0.55	-0.58	0.32	-0.24	-0.69	-0.18	0.32	-0.27	0.02
-0.12	-0.66	-0.77	0.04	-0.28	0.93	-0.13	-2.15	-0.51	-0.48	-0.42	0.08	-1.47	-0.13
0.66	0.94	0.5	1.01	-0.61	0.86	0.66	1.61	3.17	1.17	2.18	1.63	2.55	-0.2
0.64	0.53	0.99	0.94	0.11	0.51	-0.84	0.03	-0.02	0.05	-0.04	0.57	-0.27	-0.31
1.42	0.7	1.53	0.58	1.03	0.78	1.16	-0.37	2.82	0.91	-0.23	1.48	1.49	-2.1
-0.86	0.12	1.41	1.31	0.04	0.39	-1.87	0.98	-0.55	-0.51	-0.33	-0.08	-0.45	-0.89
-0.17	0.88	-0.61	0.4	0.51	0.77	0.22	1.37	-0.01	1.18	-0.04	-0.03	0.29	-1.6
0.05	-0.12	0.5	0.11	1.04	-0.02	-0.69	-0.18	-0.41	0.54	-0.14	0.12	-0.02	0.95
0.26	0.12	0.36	-1.07	0.58	0.29	0.22	0.79	-0.26	0.64	-0.02	0	-0.39	-0.15
-0.79	1.24	-0.11	0.05	-0.53	0.73	-1.27	0.97	-0.74	-0.72	-0.58	-0.03	0.01	1.05
-0.82	1.63	1.08	-0.07	0.68	0.6	-1.45	0.73	-0.08	-0.75	-1.07	0.92	-0.39	1.62
-0.01	-0.29	0.24	0.63	-0.41	0.58	-0.86	-0.52	-0.22	0.41	-0.01	0.43	0.25	0.89
0.01	0.41	-0.1	-0.57	-0.03	0.24	0.32	-0.39	-0.48	-0.22	0.08	-0.64	-0.39	0.16
-0.23	1.79	-0.41	-0.21	0.37	0.93	1.37	0.91	1.09	0.82	1.26	1.1	1.67	-0.56

L-Cysteine	Beta-Alanine	Glycerol	L-Leucine	L-Histidine	L-Proline	L-Asparagine	Citric acid	L-Valine	Cholesterol	L-Threonine	Arachidonic acid	D-Glucose	Ketoleucine
1.73	-0.46	-0.13	0.51	-0.24	-1	1.29	-1.03	0.3	-0.36	0.79	0.26	-1.09	0.6
1.45	0.44	-0.24	0.08	0.56	-1.16	0.66	-0.33	0.98	-0.87	-0.4	0.62	0	1.59
1.09	-1.11	-0.98	2.07	0.93	1.69	1.66	-1.8	2.27	-0.44	0.34	-1.36	0.67	0.18
1.49	-1.1	-0.06	-0.29	0.63	-0.67	0.19	-0.73	0.44	-1.33	1.27	-0.33	1.85	1.39
1.18	-0.37	0.09	-0.13	-0.55	-0.8	0.05	0.41	0.68	-0.03	0.47	0.15	0.67	1.05
-0.32	0.48	0.1	-0.86	0.52	-1	-0.61	-0.15	-0.63	0.31	-2.89	0.11	0.73	0.48
-0.81	-1.71	-0.02	1	0.18	0.65	-0.34	-0.03	1.32	-0.77	-0.11	0.89	-0.37	0.61
-0.87	1.06	0.98	-0.2	0.23	-0.43	-0.72	-0.23	0.45	-0.02	-0.39	-0.04	-0.01	-0.07
0.06	0.05	-2.78	-0.58	0.2	-1.66	-0.37	1.02	-1.11	1.56	-0.23	1.38	1.23	0.6
-0.26	1.33	-0.43	-0.88	1.68	1.83	0.74	1.92	-1.21	0.45	0.86	-0.41	0.05	-0.34
-0.98	0.97	0.52	0.77	-1.55	-0.07	-1.62	-1.54	0.84	-0.51	0.44	0.5	-0.74	-0.75
0.32	-1.05	0.37	0.07	1.9	2.65	2.19	1.16	-1.11	0.3	1.46	0.74	-0.47	-1.26
-0.44	-0.46	0.63	-1.38	0.38	0.19	-1.51	-1.15	-0.73	-0.08	-0.52	-0.62	0.16	-0.85
-0.04	-0.78	0.42	-0.05	0.97	-0.99	-0.27	0.38	-0.43	1.09	0.86	0.4	-1.74	-0.34
0.11	1.43	0.54	0.89	0.34	-0.63	0.09	-0.76	0.36	-0.22	0.05	-0.26	-0.84	0.36
0.15	0.21	-0.66	0.52	-0.55	-0.14	-0.96	-1.5	0.4	-0.33	-0.67	-1.35	0.86	0.15
-1.66	0.62	0.82	0.23	-2.12	0.07	-1.17	-0.91	-0.25	-1.94	0.39	-0.82	-0.52	-0.1
-1.42	1.91	0.38	0.96	-1.33	0.24	-0.39	1.14	0.1	-0.58	-0.56	-0.15	-1.22	0.05
-0.52	1.05	0.87	0.65	0.1	0.24	0.14	0.41	-0.11	-0.3	-0.56	1.11	-2.11	-0.98
0.18	-0.75	-0.88	-1.63	0.29	-0.17	-0.76	1.06	-0.73	1.52	-0.44	0.42	0.27	0.25
0.03	-0.61	-4.95	-0.5	1.17	1.22	1.18	0.84	-0.57	0.91	1.19	-0.76	-0.38	-2.19

Taurine	Palmitic acid	Citrulline	Uric acid	L-Isoleucine	N-Acetylornithine	Isocitric acid	N-Acetylglutamic acid	Malate	Oleic acid	Creatinine	Indoleacetic acid
0.66	0.21	0.47	0.37	0.59	1.2	-1.51	0.59	0.47	0.16	0.47	0.5
0.4	0.19	-0.79	0.12	0.17	-1.82	-0.59	0.16	-0.16	0.88	0.84	0.42
1.11	-1.56	-0.71	0.31	1.35	-1.9	-1.16	-2.63	-0.38	-0.41	0.23	0.1
0.44	-0.31	-0.47	1.09	-0.42	-0.08	-1.43	1.57	-0.03	0.54	-0.05	0.93
-0.26	0.43	-0.76	0.92	-0.1	0.61	-0.67	0.16	-0.56	0.85	-0.08	1.32
0.18	0.47	0.23	0.6	-0.74	-1.84	0.16	-0.51	-0.98	-0.44	0.85	-0.26
0.54	-1.11	0.52	1.6	0.31	0.07	0.1	0.11	-0.5	-0.13	0.86	0.61
0.4	0.88	0.08	0.26	-0.32	0.33	0.01	-1.19	-0.49	1.31	0.41	-0.43
0.36	0.94	-0.87	0.07	-0.84	0.3	1.09	-0.47	-0.38	1.11	0.44	-0.98
-2	-2.75	0.01	0.64	-0.35	-0.63	2.17	-0.02	1.12	-2.35	0.86	1.14
0.95	-0.44	0.78	0.85	0.48	1.91	-0.87	0.21	-0.55	-0.51	-0.23	0.31
-0.05	0.11	0.53	-1.25	-0.46	-0.32	0.97	-0.22	0.73	0.75	0.5	-1.54
0.58	0.3	-0.02	-0.17	-0.64	0.61	-0.65	-0.79	0.11	0.55	-3.3	0.05
0.21	-0.95	-1.71	-0.96	-0.9	0.79	0.24	0.12	1.52	0.16	0.46	-0.95
0.49	0.17	1.8	0.17	0.78	1.82	-0.66	-0.13	-0.24	-0.74	0.44	-2.11
-3.3	-0.09	-0.45	-1.09	0.41	0.75	-1.4	0.76	-0.88	0.2	-0.16	0.66
0.92	1.04	0.69	-1.45	0.72	-0.04	0.11	1.02	-0.66	0.3	-1.53	-0.79
0.75	-0.24	-0.28	-0.39	1.25	-0.62	0.94	1.02	-0.03	-1.09	0.57	-0.58
-0.24	-0.84	-0.94	0.12	0.55	1.32	0.69	-0.7	0.16	-0.61	-0.02	-1.37
0.16	-1.11	-1.44	-0.39	-0.99	-0.25	1.12	0.22	-0.01	-0.27	0.34	0.03
-0.82	-0.84	-0.19	-0.95	-0.59	-1.12	-1.32	-0.03	-0.27	-0.13	0.04	-0.04

g-Hydroxybutyric Acid	3-Hydroxybutyric acid	4-Hydroxyproline	Cystine	Lactic acid	Stearic acid	Capric acid	Linoleic acid	Pelargonic acid	Threonic acid	Pyroglutamic acid
0.33	-1.14	-0.02	0.1	1.65	0.78	-0.3	0.3	-0.21	1.07	0.91
-1.77	1.23	-0.94	0.15	-0.55	-0.06	0.21	1.02	-1.3	1.32	-0.4
-1.47	-0.83	-0.45	-0.46	0.89	-0.94	-0.15	0.31	-1.45	0.86	1.2
0.43	-1.18	0.08	1.54	0.52	-0.44	0.09	0.62	-2.36	2.11	1.48
0.49	1.63	-0.46	1.07	-0.05	0.51	-1.95	0.49	-0.39	1.68	-0.24
0.31	0.06	-0.51	0.73	-1.49	0.52	-0.09	0.39	-0.72	0.36	-1.25
-0.49	-0.29	0.2	-0.28	0.27	-1.2	-0.56	0.47	-0.68	-0.4	0
0.85	0.86	-2.15	-1.25	-2.17	0.08	0.36	1.03	0.48	0.19	-2.27
0.51	0.52	-0.05	0.37	-0.29	-0.03	0.87	0.23	0.64	-2.23	-0.87
-0.55	-0.54	1.83	0.24	0.2	-2.49	-0.74	0.25	-0.37	0.37	0.12
1.19	-0.29	-1	-0.92	-0.38	-0.27	-0.42	-0.25	-0.6	0.67	-0.15
-1.58	0.11	1.68	3.03	-0.32	-1.36	0.62	0.96	0.77	-0.32	0.17
-0.9	1.23	-0.85	-1.11	2.24	0.36	-0.84	0.85	-1.11	0.23	-2.25
-0.81	-0.4	0.16	-0.46	1.15	-1.7	0.23	-0.87	-0.42	-0.98	0.19
1.36	-0.46	-1.26	-0.85	-0.02	0.93	-0.31	-0.2	0.5	0.74	-0.46
0.65	-0.94	0.15	0.59	0.03	0.73	-1.73	-0.24	-0.5	-0.41	0.7
0.47	1.13	0.89	-1.82	0.11	1.36	0.47	0.12	0.96	-0.72	0.51
-1.21	-0.56	0.19	-0.74	1.04	-0.04	0.33	-1.62	1.28	0.16	2.42
1.3	-0.37	0.51	-0.68	0.44	-0.57	-0.29	0.11	-0.48	0.12	-1.31
-0.67	-0.02	0.11	0.61	0.05	-1.09	-0.15	-1.12	0.17	0.14	0.22
-2.1	-0.61	0.68	1.95	-0.84	-0.93	0.89	-0.69	0.63	0.13	0.81

1-Acylglycerol	Indolelactic acid	g-Tocopherol	Dodecanoic acid	N-Methylalanine	Glycerolphosphate	3-Aminoisobutanoic acid	5-Methoxytryptamine	2-Hydroxybutyric acid
-0.23	-0.54	0.11	0.17	1.4	1.62	-1.17	-0.14	-0.11
-1.65	0.56	0.3	0.04	0.73	0.93	0.26	0.75	1.28
-0.04	0.5	0.56	0.69	0.1	1.87	-0.53	-1.47	-1.7
-1.45	0.81	-0.8	0.2	0.64	1.15	-1.49	1.5	-0.81
-2.1	0.55	1.76	-0.19	2.19	1.25	-0.03	1.21	0.2
0.06	0.26	1.82	-3	-0.99	0.75	-0.37	1.92	0.25
-0.9	0.11	1.08	-0.6	-0.08	-3.67	-3.19	1.1	-0.47
-0.33	-0.94	-0.45	0.16	0.44	-0.75	1.02	-0.05	1.54
-0.98	0.35	0.9	1.4	-0.84	0.51	0.61	0.94	0.16
-0.5	3.17	-1.41	-3.42	-1.12	-0.76	-0.04	-0.18	-1.18
1.89	-0.1	0.83	-1.04	0.54	-0.93	1.49	-1.12	-0.27
-1.6	-1.05	0.19	0.81	0.21	-1.42	1.43	-0.15	0.2
-0.72	-0.3	-1.33	0.84	0.61	0.7	0.5	0.22	0.57
0.36	0.59	0.5	0.33	-0.02	-0.09	-0.64	0.26	-0.11
-0.25	0.14	0.5	-1.27	1	-0.96	0.45	-1.38	0.4
-0.45	-0.04	0.02	-0.03	0.52	1.49	-0.53	1.15	-0.68
2.24	-0.9	-0.71	0.75	0.94	-0.31	0.98	-0.75	-0.29
1.26	-0.95	-1.08	-0.21	-0.24	-1.04	0.81	-1.33	-2.19
1.38	-0.12	0.41	-0.71	-0.21	-0.08	-0.41	0.26	-0.17
-0.34	-0.43	0.87	-0.14	-0.29	0.84	-0.06	1.25	0.35
-1.03	0.69	0.91	0.72	0.64	-0.34	0.62	-0.21	0.85

Myristic acid	Arachidic acid	Palmitoleic acid	Pentadecanoic acid
-0.81	0.62	-0.55	-3.71
0.68	-0.02	0.36	0.41
-0.14	-0.97	-0.66	0.44
0.16	-1.39	0.78	1.29
0.87	0.16	0.69	0.87
-0.56	0.3	-1.81	0.44
-0.61	-1.32	-0.41	-0.45
0.61	-0.58	1.05	0.16
0.81	-0.82	1.17	1.04
-1.83	-2.51	-1.53	-0.12
-0.48	-0.82	0.3	-0.37
0.73	-1.24	1.44	0
0.43	0.55	0.45	0.53
0.1	-1.03	0.69	-0.79
-0.93	0.74	-0.15	-0.3
0.08	1.15	-0.16	-3.69
0.58	1.91	0.39	0.49
-0.39	1.52	-2.54	-0.44
-0.65	-0.07	-0.11	-1.15
-0.22	-0.76	-0.17	0.18
0.2	-0.33	0.52	0.06

Supplementary Table S3. Results from Visual Rule Extraction algorithm

Subject ID	Diagnosis	Predicted	Sex	Age	BMI	mtDNA CN	mtDNA deletions	L/P	BCAA/AAA	CP	Total BR	CysCys/Total Cys	Pyruvic acid	L-Glutamic acid
K10No_WD	Control	WD	2	52	-0.51	0.71	1.09	-0.19	1.42	1.45	-0.2	-1.39	0.92	-0.33
K6No_WD	Control	Control	2	38	-1.62	0.98	-0.58	-1.48	1.41	1.06	-1.08	-1.09	1	-1.84
M2No_WD	Control	WD	1	45	1.53	0.79	0.06	0.12	1.27	1.17	-1.42	-1.27	0.46	-0.08
K1No_WD	Control	Control	2	38	-1.16	0.72	-0.34	-0.38	0.96	0.69	-0.62	0.79	0.59	-2.02
K4No_WD	Control	Control	2	31	-0.39	0.26	-1.02	-0.84	1.13	0.89	-0.32	0.45	0.69	-1.86
2275WD	WD	WDNEU	2	17	-0.57	0.87	-0.81	-1.25	0.39	-0.61	-0.78	0.83	0.33	-0.61
2251WD	WD	WD	1	21	1.09	0.47	1.18	0.51	0.35	-0.86	-0.88	0.48	-0.38	0.81
1715WD	WD	WDNEU	2	30	-0.8	0.38	-1.7	-0.4	-0.01	0.3	0.02	-0.89	-0.8	0.21
1776WD	WD	WDNEU	2	39	0.29	-2.43	-0.66	0.29	0.29	0.08	0.34	0.44	-0.51	0.26
1779WD-H	WDHEP	WDHEP	1	29	1.31	-1.38	-0.8	0.11	-2.51	-0.33	1.98	0.98	0.14	0.54
2086WD-H	WDHEP	WD	2	32	-0.39	-0.2	1.27	0.3	0.41	-0.7	0.02	-0.08	-0.48	0.86
2415WD-H	WDHEP	WDHEP	1	46	0.53	-1.16	-0.21	-1.37	-1.58	0.46	2.66	1.69	1.24	0.77
972WD-H	WDHEP	WD	2	18	0.86	-1.09	-0.2	0.72	-0.01	-2.05	-0.68	-0.8	0.5	1.3
1843WD-H	WDHEP	WDNEU	2	31	0.44	-0.88	-0.31	0.23	-0.6	0.18	-0.11	-0.53	0.23	0.33
2051WD-H	WDHEP	WD	2	56	-0.83	1.3	0.67	1.12	0.38	0.61	-0.05	-1.01	-1.04	0.82
205WD-N	WDNEU	WDNEU	1	44	-1.43	0.14	0.73	-1.03	0.48	-1.71	-0.71	0.74	0.98	0.02
1167WD-N	WDNEU	WD	1	28	-1.22	-0.63	0.87	1.79	0.12	-1.43	1.01	-0.77	-1.62	0.58
1998WD-N	WDNEU	WD	1	27	-1.17	1.04	0.35	0.52	0.49	-2.79	1.49	0.67	0.13	0.06
2024WD-N	WDNEU	WDHEP	1	52	2.84	1.78	1.26	1.45	-0.2	-0.14	0.06	-0.22	-1.2	0.88
2508WD-N	WDNEU	WDNEU	2	23	-0.35	-0.32	1.61	-0.23	-0.2	0.15	-0.41	0.62	0.17	-0.09
1783WD-N	WDNEU	WDHEP	2	46	-0.38	1.67	1.05	-0.08	-1.79	-0.08	0.57	1.35	-0.36	0.28

Oxoglutaric acid	Glycine	L-Alanine	Succinic acid	L-Lysine	L-Aspartic acid	L-Glutamine	L-Serine	L-Methionine	Ornithine	L-Tryptophan	L-Phenylalanine	L-Tyrosine	Urea
-0.77	-1.01	-0.48	-0.21	-1.32	0.15	0.56	0.67	-0.25	1.29	-1.15	-0.87	-1.64	-0.72
-0.43	-0.56	-0.24	-1.55	0.66	-1.7	0.88	-1.75	-0.37	-1.53	0.24	-1.83	-1.24	0.76
-0.33	-0.66	0.53	-0.19	1.02	0.03	0.35	0.83	-0.23	0.45	1.03	-0.16	0.08	1.44
-0.72	0.36	-0.09	0.36	0.28	-1.92	0.47	-1.03	-0.44	-0.32	0.87	-1.4	-0.84	-0.48
-0.74	-1.52	-1.06	-2.42	0.46	-1.67	0.67	-0.53	-0.44	-0.24	0.51	-1.84	-0.92	-0.71
0.13	-0.72	-0.65	-0.29	-2.45	-0.19	0.52	-1	-1.45	-0.64	0.21	0.05	-1.51	1.35
-1.02	0.33	1.13	0.83	0.72	0.05	-0.2	0.85	-0.28	0.31	0.84	0.73	-0.1	-1.47
0.58	-0.43	-0.41	1.05	0.29	0.55	-0.58	0.32	-0.24	-0.69	-0.18	0.32	-0.27	0.02
-0.12	-0.66	-0.77	0.04	-0.28	0.93	-0.13	-2.15	-0.51	-0.48	-0.42	0.08	-1.47	-0.13
0.66	0.94	0.5	1.01	-0.61	0.86	0.66	1.61	3.17	1.17	2.18	1.63	2.55	-0.2
0.64	0.53	0.99	0.94	0.11	0.51	-0.84	0.03	-0.02	0.05	-0.04	0.57	-0.27	-0.31
1.42	0.7	1.53	0.58	1.03	0.78	1.16	-0.37	2.82	0.91	-0.23	1.48	1.49	-2.1
-0.86	0.12	1.41	1.31	0.04	0.39	-1.87	0.98	-0.55	-0.51	-0.33	-0.08	-0.45	-0.89
-0.17	0.88	-0.61	0.4	0.51	0.77	0.22	1.37	-0.01	1.18	-0.04	-0.03	0.29	-1.6
0.05	-0.12	0.5	0.11	1.04	-0.02	-0.69	-0.18	-0.41	0.54	-0.14	0.12	-0.02	0.95
0.26	0.12	0.36	-1.07	0.58	0.29	0.22	0.79	-0.26	0.64	-0.02	0	-0.39	-0.15
-0.79	1.24	-0.11	0.05	-0.53	0.73	-1.27	0.97	-0.74	-0.72	-0.58	-0.03	0.01	1.05
-0.82	1.63	1.08	-0.07	0.68	0.6	-1.45	0.73	-0.08	-0.75	-1.07	0.92	-0.39	1.62
-0.01	-0.29	0.24	0.63	-0.41	0.58	-0.86	-0.52	-0.22	0.41	-0.01	0.43	0.25	0.89
0.01	0.41	-0.1	-0.57	-0.03	0.24	0.32	-0.39	-0.48	-0.22	0.08	-0.64	-0.39	0.16
-0.23	1.79	-0.41	-0.21	0.37	0.93	1.37	0.91	1.09	0.82	1.26	1.1	1.67	-0.56

L-Cysteine	b-Alanine	Glycerol	L-Leucine	L-Histidine	L-Proline	L-Asparagine	Citric acid	L-Valine	Cholesterol	L-Threonine	Arachidonic acid	D-Glucose	Ketoleucine	Taurine
1.73	-0.46	-0.13	0.51	-0.24	-1	1.29	-1.03	0.3	-0.36	0.79	0.26	-1.09	0.6	0.66
1.45	0.44	-0.24	0.08	0.56	-1.16	0.66	-0.33	0.98	-0.87	-0.4	0.62	0	1.59	0.4
1.09	-1.11	-0.98	2.07	0.93	1.69	1.66	-1.8	2.27	-0.44	0.34	-1.36	0.67	0.18	1.11
1.49	-1.1	-0.06	-0.29	0.63	-0.67	0.19	-0.73	0.44	-1.33	1.27	-0.33	1.85	1.39	0.44
1.18	-0.37	0.09	-0.13	-0.55	-0.8	0.05	0.41	0.68	-0.03	0.47	0.15	0.67	1.05	-0.26
-0.32	0.48	0.1	-0.86	0.52	-1	-0.61	-0.15	-0.63	0.31	-2.89	0.11	0.73	0.48	0.18
-0.81	-1.71	-0.02	1	0.18	0.65	-0.34	-0.03	1.32	-0.77	-0.11	0.89	-0.37	0.61	0.54
-0.87	1.06	0.98	-0.2	0.23	-0.43	-0.72	-0.23	0.45	-0.02	-0.39	-0.04	-0.01	-0.07	0.4
0.06	0.05	-2.78	-0.58	0.2	-1.66	-0.37	1.02	-1.11	1.56	-0.23	1.38	1.23	0.6	0.36
-0.26	1.33	-0.43	-0.88	1.68	1.83	0.74	1.92	-1.21	0.45	0.86	-0.41	0.05	-0.34	-2
-0.98	0.97	0.52	0.77	-1.55	-0.07	-1.62	-1.54	0.84	-0.51	0.44	0.5	-0.74	-0.75	0.95
0.32	-1.05	0.37	0.07	1.9	2.65	2.19	1.16	-1.11	0.3	1.46	0.74	-0.47	-1.26	-0.05
-0.44	-0.46	0.63	-1.38	0.38	0.19	-1.51	-1.15	-0.73	-0.08	-0.52	-0.62	0.16	-0.85	0.58
-0.04	-0.78	0.42	-0.05	0.97	-0.99	-0.27	0.38	-0.43	1.09	0.86	0.4	-1.74	-0.34	0.21
0.11	1.43	0.54	0.89	0.34	-0.63	0.09	-0.76	0.36	-0.22	0.05	-0.26	-0.84	0.36	0.49
0.15	0.21	-0.66	0.52	-0.55	-0.14	-0.96	-1.5	0.4	-0.33	-0.67	-1.35	0.86	0.15	-3.3
-1.66	0.62	0.82	0.23	-2.12	0.07	-1.17	-0.91	-0.25	-1.94	0.39	-0.82	-0.52	-0.1	0.92
-1.42	1.91	0.38	0.96	-1.33	0.24	-0.39	1.14	0.1	-0.58	-0.56	-0.15	-1.22	0.05	0.75
-0.52	1.05	0.87	0.65	0.1	0.24	0.14	0.41	-0.11	-0.3	-0.56	1.11	-2.11	-0.98	-0.24
0.18	-0.75	-0.88	-1.63	0.29	-0.17	-0.76	1.06	-0.73	1.52	-0.44	0.42	0.27	0.25	0.16
0.03	-0.61	-4.95	-0.5	1.17	1.22	1.18	0.84	-0.57	0.91	1.19	-0.76	-0.38	-2.19	-0.82

Palmitic acid	Citrulline	Uric acid	L-Isoleucine	N-Acetylornithine	Isocitric acid	N-Acetylglutamic acid	Malate	Oleic acid	Creatinine	Indoleacetic acid	g-Hydroxybutyric Acid
0.21	0.47	0.37	0.59	1.2	-1.51	0.59	0.47	0.16	0.47	0.5	0.33
0.19	-0.79	0.12	0.17	-1.82	-0.59	0.16	-0.16	0.88	0.84	0.42	-1.77
-1.56	-0.71	0.31	1.35	-1.9	-1.16	-2.63	-0.38	-0.41	0.23	0.1	-1.47
-0.31	-0.47	1.09	-0.42	-0.08	-1.43	1.57	-0.03	0.54	-0.05	0.93	0.43
0.43	-0.76	0.92	-0.1	0.61	-0.67	0.16	-0.56	0.85	-0.08	1.32	0.49
0.47	0.23	0.6	-0.74	-1.84	0.16	-0.51	-0.98	-0.44	0.85	-0.26	0.31
-1.11	0.52	1.6	0.31	0.07	0.1	0.11	-0.5	-0.13	0.86	0.61	-0.49
0.88	0.08	0.26	-0.32	0.33	0.01	-1.19	-0.49	1.31	0.41	-0.43	0.85
0.94	-0.87	0.07	-0.84	0.3	1.09	-0.47	-0.38	1.11	0.44	-0.98	0.51
-2.75	0.01	0.64	-0.35	-0.63	2.17	-0.02	1.12	-2.35	0.86	1.14	-0.55
-0.44	0.78	0.85	0.48	1.91	-0.87	0.21	-0.55	-0.51	-0.23	0.31	1.19
0.11	0.53	-1.25	-0.46	-0.32	0.97	-0.22	0.73	0.75	0.5	-1.54	-1.58
0.3	-0.02	-0.17	-0.64	0.61	-0.65	-0.79	0.11	0.55	-3.3	0.05	-0.9
-0.95	-1.71	-0.96	-0.9	0.79	0.24	0.12	1.52	0.16	0.46	-0.95	-0.81
0.17	1.8	0.17	0.78	1.82	-0.66	-0.13	-0.24	-0.74	0.44	-2.11	1.36
-0.09	-0.45	-1.09	0.41	0.75	-1.4	0.76	-0.88	0.2	-0.16	0.66	0.65
1.04	0.69	-1.45	0.72	-0.04	0.11	1.02	-0.66	0.3	-1.53	-0.79	0.47
-0.24	-0.28	-0.39	1.25	-0.62	0.94	1.02	-0.03	-1.09	0.57	-0.58	-1.21
-0.84	-0.94	0.12	0.55	1.32	0.69	-0.7	0.16	-0.61	-0.02	-1.37	1.3
-1.11	-1.44	-0.39	-0.99	-0.25	1.12	0.22	-0.01	-0.27	0.34	0.03	-0.67
-0.84	-0.19	-0.95	-0.59	-1.12	-1.32	-0.03	-0.27	-0.13	0.04	-0.04	-2.1

3-Hydroxybutyric acid	4-Hydroxyproline	Cystine	Lactic acid	Stearic acid	Capric acid	Linoleic acid	Pelargonic acid	Threonic acid	Pyroglutamic acid	1-Acylglycerol
-1.14	-0.02	0.1	1.65	0.78	-0.3	0.3	-0.21	1.07	0.91	-0.23
1.23	-0.94	0.15	-0.55	-0.06	0.21	1.02	-1.3	1.32	-0.4	-1.65
-0.83	-0.45	-0.46	0.89	-0.94	-0.15	0.31	-1.45	0.86	1.2	-0.04
-1.18	0.08	1.54	0.52	-0.44	0.09	0.62	-2.36	2.11	1.48	-1.45
1.63	-0.46	1.07	-0.05	0.51	-1.95	0.49	-0.39	1.68	-0.24	-2.1
0.06	-0.51	0.73	-1.49	0.52	-0.09	0.39	-0.72	0.36	-1.25	0.06
-0.29	0.2	-0.28	0.27	-1.2	-0.56	0.47	-0.68	-0.4	0	-0.9
0.86	-2.15	-1.25	-2.17	0.08	0.36	1.03	0.48	0.19	-2.27	-0.33
0.52	-0.05	0.37	-0.29	-0.03	0.87	0.23	0.64	-2.23	-0.87	-0.98
-0.54	1.83	0.24	0.2	-2.49	-0.74	0.25	-0.37	0.37	0.12	-0.5
-0.29	-1	-0.92	-0.38	-0.27	-0.42	-0.25	-0.6	0.67	-0.15	1.89
0.11	1.68	3.03	-0.32	-1.36	0.62	0.96	0.77	-0.32	0.17	-1.6
1.23	-0.85	-1.11	2.24	0.36	-0.84	0.85	-1.11	0.23	-2.25	-0.72
-0.4	0.16	-0.46	1.15	-1.7	0.23	-0.87	-0.42	-0.98	0.19	0.36
-0.46	-1.26	-0.85	-0.02	0.93	-0.31	-0.2	0.5	0.74	-0.46	-0.25
-0.94	0.15	0.59	0.03	0.73	-1.73	-0.24	-0.5	-0.41	0.7	-0.45
1.13	0.89	-1.82	0.11	1.36	0.47	0.12	0.96	-0.72	0.51	2.24
-0.56	0.19	-0.74	1.04	-0.04	0.33	-1.62	1.28	0.16	2.42	1.26
-0.37	0.51	-0.68	0.44	-0.57	-0.29	0.11	-0.48	0.12	-1.31	1.38
-0.02	0.11	0.61	0.05	-1.09	-0.15	-1.12	0.17	0.14	0.22	-0.34
-0.61	0.68	1.95	-0.84	-0.93	0.89	-0.69	0.63	0.13	0.81	-1.03

Indolelactic acid	Gamma-Tocopherol	Dodecanoic acid	N-Methylalanine	Glycerolphosphate	3-Aminoisobutanoic acid	5-Methoxytryptamine	2-Hydroxybutyric acid
-0.54	0.11	0.17	1.4	1.62	-1.17	-0.14	-0.11
0.56	0.3	0.04	0.73	0.93	0.26	0.75	1.28
0.5	0.56	0.69	0.1	1.87	-0.53	-1.47	-1.7
0.81	-0.8	0.2	0.64	1.15	-1.49	1.5	-0.81
0.55	1.76	-0.19	2.19	1.25	-0.03	1.21	0.2
0.26	1.82	-3	-0.99	0.75	-0.37	1.92	0.25
0.11	1.08	-0.6	-0.08	-3.67	-3.19	1.1	-0.47
-0.94	-0.45	0.16	0.44	-0.75	1.02	-0.05	1.54
0.35	0.9	1.4	-0.84	0.51	0.61	0.94	0.16
3.17	-1.41	-3.42	-1.12	-0.76	-0.04	-0.18	-1.18
-0.1	0.83	-1.04	0.54	-0.93	1.49	-1.12	-0.27
-1.05	0.19	0.81	0.21	-1.42	1.43	-0.15	0.2
-0.3	-1.33	0.84	0.61	0.7	0.5	0.22	0.57
0.59	0.5	0.33	-0.02	-0.09	-0.64	0.26	-0.11
0.14	0.5	-1.27	1	-0.96	0.45	-1.38	0.4
-0.04	0.02	-0.03	0.52	1.49	-0.53	1.15	-0.68
-0.9	-0.71	0.75	0.94	-0.31	0.98	-0.75	-0.29
-0.95	-1.08	-0.21	-0.24	-1.04	0.81	-1.33	-2.19
-0.12	0.41	-0.71	-0.21	-0.08	-0.41	0.26	-0.17
-0.43	0.87	-0.14	-0.29	0.84	-0.06	1.25	0.35
0.69	0.91	0.72	0.64	-0.34	0.62	-0.21	0.85

Myristic acid	Arachidic acid	Palmitoleic acid	Pentadecanoic acid
-0.81	0.62	-0.55	-3.71
0.68	-0.02	0.36	0.41
-0.14	-0.97	-0.66	0.44
0.16	-1.39	0.78	1.29
0.87	0.16	0.69	0.87
-0.56	0.3	-1.81	0.44
-0.61	-1.32	-0.41	-0.45
0.61	-0.58	1.05	0.16
0.81	-0.82	1.17	1.04
-1.83	-2.51	-1.53	-0.12
-0.48	-0.82	0.3	-0.37
0.73	-1.24	1.44	0
0.43	0.55	0.45	0.53
0.1	-1.03	0.69	-0.79
-0.93	0.74	-0.15	-0.3
0.08	1.15	-0.16	-3.69
0.58	1.91	0.39	0.49
-0.39	1.52	-2.54	-0.44
-0.65	-0.07	-0.11	-1.15
-0.22	-0.76	-0.17	0.18
0.2	-0.33	0.52	0.06

Supplementary Table S4. PCA variance when using all outcomes

	Individual	**Cumulative**
**PC1**	0.15	**0.15**
**PC2**	0.12	**0.27**
**PC3**	0.10	**0.37**
**PC4**	0.07	**0.44**
**PC5**	0.05	**0.48**
**PC6**	0.04	**0.52**
**PC7**	0.04	**0.56**
**PC8**	0.04	**0.60**
**PC9**	0.03	**0.63**
**PC10**	0.03	**0.66**
**PC11**	0.03	**0.69**
**PC12**	0.02	**0.71**
**PC13**	0.02	**0.73**
**PC14**	0.02	**0.75**
**PC15**	0.02	**0.77**
**PC16**	0.02	**0.79**
**PC17**	0.02	**0.80**
**PC18**	0.02	**0.82**
**PC19**	0.01	**0.83**
**PC20**	0.01	**0.85**
PC21	0.01	0.86
PC22	0.01	0.87
PC23	0.01	0.88
PC24	0.01	0.89
PC25	0.01	0.90
PC26	0.01	0.91
PC27	0.01	0.91
PC28	0.01	0.92
PC29	0.01	0.93
PC30	0.01	0.94
PC31	0.01	0.94
PC32	0.01	0.95
PC33	0.00	0.95
PC34	0.00	0.96
PC35	0.00	0.96
PC36	0.00	0.97
PC37	0.00	0.97
PC38	0.00	0.97
PC39	0.00	0.98
PC40	0.00	0.98

PC41	0.00	0.98
PC42	0.00	0.98
PC43	0.00	0.99
PC44	0.00	0.99
PC45	0.00	0.99
PC46	0.00	0.99
PC47	0.00	0.99
PC48	0.00	0.99
PC49	0.00	0.99
PC50	0.00	1.00
PC51	0.00	1.00
PC52	0.00	1.00
PC53	0.00	1.00
PC54	0.00	1.00
PC55	0.00	1.00
PC56	0.00	1.00
PC57	0.00	1.00
PC58	0.00	1.00
PC59	0.00	1.00
PC60	0.00	1.00
PC61	0.00	1.00
PC62	0.00	1.00

Supplementary Table S5. PCA loadings when using all outcomes

	PC1	PC2	PC3	PC4	PC5	PC6	PC7	PC8	PC9	PC10	PC11	PC12	PC13	PC14	PC15	PC16	PC17	PC18	PC19	PC20	PC21	PC22	PC23	PC24	PC25	PC26	PC27	PC28	PC29	PC30
BCAA/AAA	0.26	0.03	-0.06	0.07	-0.03	-0.11	0.09	-0.01	-0.03	0.03	0.10	0.08	-0.17	0.08	-0.05	0.00	-0.04	0.06	-0.03	0.08	-0.02	0.06	-0.01	-0.04	0.04	-0.10	0.05	-0.04	0.01	0.02
Threonic acid	0.22	-0.01	0.00	-0.02	0.07	0.03	0.09	-0.08	-0.09	-0.09	0.07	-0.07	0.18	-0.06	0.14	0.01	0.15	-0.09	-0.03	0.03	0.18	0.01	0.06	0.04	-0.16	0.19	0.15	-0.14	0.08	-0.01
Ketoleucine	0.18	0.05	0.02	0.14	0.13	-0.05	-0.07	0.06	0.17	0.18	0.00	-0.10	-0.05	0.29	0.10	-0.13	0.07	0.03	-0.19	0.01	-0.03	0.12	-0.15	-0.06	0.00	0.00	0.02	0.08	0.13	0.02
Uric acid	0.18	-0.09	0.06	0.20	-0.07	-0.06	-0.11	-0.01	0.08	-0.06	0.15	-0.01	-0.02	0.09	0.16	0.19	-0.06	0.02	0.08	0.12	0.02	-0.02	-0.07	-0.06	-0.10	0.09	0.14	0.16	-0.04	0.11
L-Cysteine	0.17	-0.14	0.13	-0.06	-0.04	-0.01	0.18	0.05	-0.11	0.04	0.00	0.00	-0.01	0.05	0.04	-0.02	-0.10	0.06	-0.13	-0.01	-0.28	-0.11	0.03	-0.04	-0.17	0.08	-0.21	-0.06	-0.04	-0.02
Glycerolphosphate	0.15	-0.02	0.09	0.01	0.05	0.11	0.11	0.06	-0.06	-0.18	-0.24	0.06	0.00	0.19	-0.08	0.10	-0.19	0.20	0.02	-0.05	0.02	0.19	0.24	0.07	-0.02	0.06	-0.14	-0.13	0.33	-0.02
D-Glucose	0.15	-0.08	0.09	-0.11	0.08	-0.14	-0.23	0.01	0.11	0.01	-0.10	-0.08	-0.10	-0.04	0.06	0.14	0.07	0.14	0.03	0.00	-0.07	-0.15	-0.13	0.01	0.16	-0.12	-0.05	-0.09	0.22	0.13
L-Valine	0.14	-0.14	-0.15	0.08	-0.12	-0.22	0.01	-0.11	-0.04	0.11	0.00	-0.03	-0.11	-0.01	-0.10	0.03	-0.02	0.04	-0.07	0.07	0.03	0.18	-0.09	0.03	0.02	-0.01	0.15	0.13	0.14	-0.13
mtDNA CN	0.13	-0.05	-0.01	-0.09	-0.12	0.09	0.19	0.04	-0.05	0.00	0.18	0.16	0.14	-0.09	-0.11	0.11	0.04	-0.15	0.14	0.04	0.37	-0.03	-0.17	-0.13	-0.02	0.13	-0.05	-0.03	0.12	-0.21
Indoleacetic acid	0.13	-0.13	-0.02	0.03	0.00	0.02	-0.14	-0.07	0.16	-0.06	-0.22	0.17	0.02	0.06	0.13	-0.09	0.07	0.04	0.22	0.12	-0.02	0.06	0.29	0.05	0.18	0.25	-0.08	-0.01	-0.01	0.08
Indolelactic acid	0.12	-0.19	0.00	0.05	0.00	0.03	-0.18	-0.09	0.12	-0.09	0.00	-0.18	0.12	-0.02	0.08	0.04	-0.06	-0.01	0.05	-0.07	-0.25	-0.10	0.03	-0.17	0.01	-0.05	0.22	0.02	0.02	-0.12
CP	0.12	-0.02	0.12	-0.11	-0.04	-0.02	0.04	-0.21	-0.14	0.11	0.13	-0.14	0.12	0.11	0.13	-0.07	-0.34	-0.17	0.10	-0.08	0.02	0.01	-0.02	0.28	0.07	-0.19	-0.01	0.19	-0.07	0.21
Creatinine	0.10	-0.13	0.04	0.13	-0.08	0.26	-0.01	0.05	-0.05	0.12	0.16	0.00	-0.09	-0.01	-0.01	0.03	0.24	-0.23	-0.04	-0.08	0.00	0.20	0.11	-0.09	-0.07	0.03	0.06	0.20	0.04	0.15
L-Glutamine	0.10	-0.15	0.08	-0.15	-0.08	0.30	0.12	0.03	-0.01	0.05	0.00	-0.07	0.08	0.04	0.01	0.07	0.08	0.08	-0.04	0.01	-0.10	-0.06	-0.08	0.08	-0.02	-0.06	0.06	0.06	-0.07	-0.04
Gamma-Tocopherol	0.09	-0.06	0.10	-0.05	-0.04	0.12	-0.02	-0.06	0.10	0.10	0.16	0.31	-0.12	-0.25	-0.17	-0.07	-0.05	0.04	0.00	-0.07	-0.15	-0.05	0.06	0.50	-0.20	0.09	0.13	-0.01	0.12	-0.15
Stearic acid	0.08	0.25	-0.13	-0.08	-0.06	0.07	-0.02	0.01	0.01	0.05	-0.13	-0.08	0.03	0.09	0.08	0.14	-0.01	0.10	-0.09	0.02	0.12	-0.05	0.07	0.01	0.03	0.00	-0.02	0.03	-0.02	0.00
Pyroglutamic acid	0.07	-0.01	-0.03	-0.30	0.14	-0.12	0.18	0.03	0.20	-0.03	0.04	0.05	0.06	-0.04	0.05	0.01	0.09	-0.05	-0.09	-0.08	0.09	0.07	0.06	-0.13	0.01	0.03	0.22	0.15	0.16	0.05
L-Leucine	0.06	-0.12	-0.19	0.08	-0.09	-0.22	0.08	-0.05	0.10	0.19	0.00	-0.02	-0.14	0.03	-0.11	0.04	0.09	-0.15	-0.03	-0.05	-0.06	0.22	-0.06	0.01	-0.06	0.04	-0.16	0.13	-0.02	-0.02
N-Acetylglutamic acid	0.06	0.08	0.00	-0.21	0.04	-0.06	0.09	0.10	0.26	0.01	0.05	0.07	0.03	0.08	-0.01	-0.17	0.16	-0.25	0.17	0.20	-0.20	-0.24	-0.16	-0.04	-0.06	0.14	-0.23	-0.03	0.02	0.20
L-Asparagine	0.06	-0.17	0.04	-0.16	-0.13	0.08	0.17	-0.09	-0.04	0.12	0.15	-0.14	0.04	0.17	0.12	0.04	0.05	0.04	0.02	-0.14	0.18	0.08	0.06	-0.01	0.12	-0.10	-0.20	-0.03	0.18	0.02
Pyruvic acid	0.05	-0.08	0.16	0.03	0.15	-0.14	0.02	0.28	-0.01	0.30	-0.03	0.02	0.16	0.01	-0.13	0.00	-0.10	0.04	-0.08	0.06	0.07	-0.13	0.13	-0.09	0.06	0.04	0.16	-0.03	-0.03	-0.01
L-Tryptophan	0.05	-0.24	-0.04	0.08	-0.05	0.07	-0.19	-0.04	0.06	-0.04	-0.13	-0.14	0.04	-0.02	0.05	0.18	0.09	0.08	0.00	-0.04	-0.11	-0.11	-0.13	-0.02	-0.08	0.18	0.02	0.12	0.02	-0.02
Urea	0.04	0.06	-0.20	-0.02	0.13	0.19	-0.12	-0.23	-0.06	0.05	-0.02	0.03	0.09	0.11	-0.18	0.02	-0.11	-0.15	-0.12	0.20	-0.06	0.05	-0.07	0.01	0.20	0.04	-0.01	-0.06	0.17	0.04
Pentadecanoic acid	0.04	0.02	0.08	-0.07	0.12	-0.01	-0.22	-0.18	-0.06	-0.02	0.26	-0.20	-0.16	-0.09	0.06	-0.04	0.13	0.26	0.08	0.07	0.13	-0.22	0.16	-0.08	-0.04	0.04	-0.30	0.22	0.22	-0.18
(R)-3-Hydroxybutyric acid	0.04	0.16	0.07	0.00	-0.05	0.10	0.04	0.01	0.11	0.10	0.09	-0.25	0.01	0.05	-0.32	-0.10	0.15	0.25	0.36	-0.12	-0.05	0.04	-0.11	-0.06	-0.05	-0.09	0.15	-0.15	0.01	-0.07
L-Isoleucine	0.03	-0.04	-0.26	-0.06	-0.14	-0.19	0.04	-0.03	0.07	0.11	0.09	-0.02	-0.13	0.16	-0.02	0.03	0.07	-0.04	-0.11	0.04	-0.11	0.09	-0.04	0.06	0.14	0.15	-0.02	-0.20	0.06	-0.19
5-Methoxytryptamine	0.03	-0.10	0.19	0.08	-0.08	0.10	-0.08	0.26	0.02	-0.15	-0.07	0.07	-0.04	0.13	-0.09	0.01	0.04	0.13	-0.01	0.18	-0.04	0.01	-0.32	0.13	-0.04	-0.09	-0.15	0.01	0.02	-0.12
N-Methylalanine	0.03	-0.02	0.04	0.00	-0.15	-0.02	0.09	-0.20	-0.11	-0.24	-0.08	0.21	0.20	0.34	-0.07	-0.27	-0.01	0.23	0.01	0.02	0.01	0.03	-0.10	-0.05	-0.04	0.12	0.20	0.10	-0.07	-0.01
Gamma Hydroxybutyric Acid	0.03	0.17	-0.16	0.01	-0.13	0.06	-0.06	0.19	0.03	0.07	-0.11	-0.04	0.01	-0.04	0.18	-0.09	-0.22	-0.07	0.18	-0.06	-0.05	0.07	-0.14	0.05	-0.10	0.02	0.05	-0.08	0.31	0.05
L-Lysine	0.03	-0.10	-0.09	0.07	-0.05	0.08	0.10	-0.07	0.11	0.27	-0.24	-0.04	-0.22	0.06	0.03	-0.20	0.08	-0.04	0.28	0.09	0.19	-0.16	-0.04	-0.04	-0.06	-0.03	0.02	-0.09	-0.20	-0.25
Taurine	0.03	-0.01	-0.05	0.24	0.12	0.14	0.01	-0.26	0.02	0.17	0.06	0.15	-0.13	0.03	0.01	-0.10	-0.11	0.22	-0.03	0.19	0.13	-0.09	0.01	-0.09	-0.11	-0.03	0.02	0.10	-0.08	0.05
Arachidic acid	0.02	0.21	-0.17	-0.12	-0.02	0.09	0.09	0.06	0.00	0.00	-0.09	-0.05	0.02	0.09	-0.06	0.19	0.08	0.04	-0.21	0.02	0.08	-0.08	0.04	0.08	0.00	0.03	-0.20	0.06	-0.13	-0.09
Lactic acid	0.01	0.02	0.06	0.19	0.12	-0.15	0.26	0.11	0.22	-0.10	0.19	-0.07	0.02	0.09	-0.11	-0.03	-0.03	0.11	-0.02	0.05	0.06	-0.17	0.31	-0.07	0.09	0.06	0.06	-0.14	-0.02	0.10
Oleic acid	0.00	0.18	0.24	0.05	-0.16	-0.11	0.04	-0.06	0.02	0.05	-0.01	-0.04	0.01	-0.04	-0.03	0.03	0.16	-0.01	-0.07	-0.04	-0.13	-0.08	-0.04	0.02	0.03	-0.06	-0.05	0.03	0.03	-0.02
Palmitic acid	0.00	0.28	0.08	-0.02	-0.15	0.02	0.02	-0.04	0.02	0.13	-0.09	-0.07	0.01	0.06	0.07	0.16	0.08	0.03	-0.05	0.10	0.05	-0.04	0.02	-0.05	0.00	-0.03	0.03	0.06	0.02	0.02
Linoleic acid	-0.01	0.10	0.20	0.12	-0.15	-0.13	-0.04	-0.08	0.10	0.04	0.07	-0.09	0.26	-0.03	-0.04	0.05	0.08	0.02	-0.17	-0.08	-0.02	0.12	-0.02	-0.17	-0.13	0.13	-0.01	-0.11	-0.11	0.04
Cystine	-0.02	-0.13	0.22	-0.17	-0.18	0.00	-0.07	0.01	0.07	0.01	-0.06	0.10	-0.07	0.12	-0.04	0.01	-0.04	-0.11	-0.13	0.14	0.10	-0.04	0.04	-0.07	-0.12	-0.06	-0.08	-0.03	-0.01	0.01

Myristic acid	-0.03	0.13	0.19	-0.09	0.01	-0.04	0.08	-0.18	0.00	0.08	-0.17	0.05	-0.02	-0.13	-0.05	0.20	0.00	0.05	-0.01	0.26	-0.17	0.10	0.02	0.00	0.04	0.15	0.13	0.04	0.11	-0.23
Beta-Alanine	-0.03	0.05	-0.21	-0.02	0.00	0.25	0.04	0.11	-0.18	0.08	0.11	-0.03	0.10	0.18	0.11	0.11	0.02	0.06	-0.06	0.13	-0.21	-0.13	0.05	-0.14	0.06	0.11	0.16	-0.07	0.07	-0.22
Capric acid	-0.03	0.04	0.07	0.01	0.22	0.27	0.15	-0.18	0.11	0.16	-0.13	0.12	-0.08	-0.07	0.03	0.04	0.04	0.02	0.02	-0.13	-0.09	0.08	-0.19	-0.26	-0.19	0.15	0.00	-0.08	0.00	0.25
Dodecanoic acid	-0.04	0.12	0.14	-0.08	0.06	-0.09	0.25	-0.23	0.02	0.11	-0.23	0.01	-0.18	-0.11	0.03	0.05	-0.02	0.08	-0.01	0.00	0.03	0.02	-0.03	-0.01	0.17	-0.09	0.00	0.03	-0.03	-0.03
Ornithine	-0.04	-0.16	-0.01	0.07	-0.15	0.23	0.29	0.10	0.11	0.11	0.02	-0.06	0.06	-0.06	0.11	-0.04	0.06	0.09	-0.03	-0.05	-0.08	-0.02	0.09	-0.01	0.03	-0.16	-0.06	0.05	0.17	-0.06
Arachidonic acid	-0.04	0.10	0.08	-0.09	-0.22	0.02	-0.11	-0.03	0.09	0.09	0.28	0.26	-0.01	-0.04	0.12	-0.14	0.10	0.10	0.00	-0.01	-0.18	0.16	0.20	-0.07	0.23	0.12	-0.03	0.04	-0.11	-0.06
L-Histidine	-0.04	-0.22	0.11	0.09	-0.06	0.16	-0.01	-0.16	-0.07	0.09	-0.03	-0.07	-0.01	0.10	0.04	0.08	-0.05	-0.05	-0.16	0.13	0.00	-0.01	0.04	0.01	0.06	0.05	-0.10	-0.14	-0.21	0.09
Cholesterol	-0.05	-0.02	0.25	0.07	-0.07	0.11	0.03	0.10	-0.05	-0.07	-0.02	0.03	-0.21	0.00	0.02	0.26	-0.10	-0.19	0.21	-0.04	0.09	-0.05	0.11	-0.06	0.06	0.18	0.00	0.03	0.02	0.01
N-Acetylornithine	-0.06	0.07	-0.10	0.10	-0.29	0.02	0.12	0.21	-0.07	-0.11	-0.06	-0.07	-0.06	-0.09	0.22	-0.24	-0.08	0.02	0.08	0.14	-0.01	-0.06	-0.01	-0.11	0.08	-0.08	0.09	0.15	-0.02	-0.02
L/P	-0.06	0.08	-0.14	0.08	-0.08	0.05	0.12	-0.25	0.14	-0.36	0.13	-0.07	-0.16	0.06	0.07	0.00	0.09	0.03	0.09	-0.02	-0.03	0.03	0.03	0.06	-0.01	0.00	-0.13	-0.06	0.03	0.06
Citrulline	-0.06	0.07	-0.21	-0.08	-0.08	0.01	-0.15	-0.01	0.03	0.22	-0.05	0.05	0.07	-0.07	0.21	-0.01	-0.04	0.17	-0.10	-0.04	0.23	-0.16	0.09	0.14	-0.26	0.14	0.08	-0.08	0.00	0.13
mtDNA deletions	-0.06	-0.03	-0.12	-0.07	-0.24	0.05	0.09	0.00	0.28	-0.09	0.03	0.12	0.02	-0.04	-0.11	0.05	-0.24	0.05	-0.23	0.09	-0.02	-0.15	-0.08	-0.11	-0.10	-0.10	-0.05	0.17	0.12	0.09
1-Acylglycerol	-0.07	0.10	-0.16	0.07	0.17	0.01	-0.01	-0.01	0.13	0.05	0.04	0.21	0.25	0.10	-0.03	0.15	-0.11	-0.03	0.25	-0.13	-0.15	-0.02	0.07	-0.11	-0.01	-0.03	-0.17	0.39	-0.01	-0.13
Palmitoleic acid	-0.07	0.14	0.22	0.03	-0.18	-0.10	0.08	-0.11	-0.02	0.02	-0.04	-0.08	0.07	-0.01	0.14	-0.03	-0.02	0.05	-0.05	-0.07	-0.03	-0.13	-0.14	0.06	0.02	0.12	0.05	0.20	0.12	-0.03
Glycerol	-0.07	0.12	0.03	0.09	-0.04	-0.07	0.05	0.02	-0.05	0.12	0.11	-0.01	0.27	-0.05	0.16	0.14	0.11	0.11	0.22	0.44	-0.06	0.33	0.00	0.15	-0.20	-0.09	-0.13	-0.08	0.00	0.11
Malate	-0.08	0.02	0.11	0.05	0.21	-0.02	0.14	0.02	0.21	-0.13	-0.06	-0.26	-0.04	0.15	0.06	-0.04	-0.20	-0.18	-0.03	0.09	0.04	0.18	0.06	0.08	-0.23	0.07	0.00	0.16	-0.11	-0.37
L-Alanine	-0.08	-0.17	-0.06	0.07	0.08	-0.24	0.04	0.17	-0.16	-0.01	0.12	0.15	-0.01	-0.01	0.00	0.09	0.05	0.18	-0.02	0.04	0.11	-0.10	-0.13	0.05	-0.12	0.07	-0.12	0.05	-0.08	-0.05
2-Hydroxybutyric acid	-0.09	0.14	0.12	0.14	-0.06	0.05	-0.13	-0.03	-0.03	0.06	0.02	-0.03	0.05	0.24	-0.17	-0.28	0.04	-0.03	-0.12	-0.22	0.15	-0.04	-0.09	0.11	-0.03	0.23	-0.09	0.01	0.17	0.02
L-Threonine	-0.10	-0.17	-0.01	-0.14	-0.09	-0.18	0.16	0.00	-0.15	0.07	-0.14	0.00	-0.14	-0.05	0.18	-0.11	-0.05	0.11	0.06	-0.02	-0.04	0.04	0.00	-0.13	-0.03	0.21	0.06	0.09	0.10	0.11
Succinic acid	-0.10	0.00	0.08	0.17	0.07	-0.11	0.02	-0.17	-0.03	-0.09	0.13	0.17	0.00	0.02	0.35	0.03	-0.06	-0.17	-0.01	-0.07	-0.01	-0.07	-0.16	-0.12	-0.09	-0.13	0.01	-0.37	0.21	-0.21
Pelargonic acid	-0.10	0.11	0.01	-0.01	0.01	-0.01	0.08	0.05	0.09	0.03	0.18	0.09	-0.27	0.36	0.12	0.34	0.02	-0.02	0.08	-0.09	-0.02	-0.18	-0.11	0.25	0.07	0.13	0.23	0.01	-0.09	0.11
3-Aminoisobutanoic acid	-0.11	0.03	-0.02	-0.13	-0.07	-0.12	-0.02	-0.05	-0.37	0.02	0.03	-0.05	-0.12	0.16	-0.29	0.09	-0.10	-0.02	0.22	-0.01	-0.08	-0.02	0.04	-0.25	-0.20	0.09	0.01	0.06	0.11	0.11
BMI	-0.11	-0.05	-0.01	0.08	-0.07	0.06	0.06	-0.04	0.22	0.05	0.20	-0.20	0.06	-0.22	-0.13	-0.01	-0.42	0.07	0.02	0.11	0.07	-0.01	-0.16	-0.04	0.26	0.30	-0.06	-0.11	0.03	0.04
L-Proline	-0.12	-0.20	-0.06	-0.15	0.00	-0.04	0.05	-0.16	0.01	0.02	0.06	0.01	0.16	0.05	-0.06	0.14	-0.02	0.19	0.16	-0.02	0.01	0.01	-0.15	0.04	0.11	-0.11	0.04	-0.09	-0.15	0.06
L-Serine	-0.12	-0.16	-0.05	0.13	0.11	-0.05	0.20	0.05	0.09	-0.08	-0.11	-0.08	0.12	0.01	-0.03	0.05	0.20	0.11	0.06	0.01	0.10	0.05	-0.04	0.23	0.07	0.03	0.08	0.11	0.16	0.19
CysCys/Total Cys	-0.13	-0.06	0.11	-0.10	-0.18	0.03	-0.24	-0.01	0.18	-0.07	-0.08	0.10	-0.01	0.10	-0.07	0.04	0.07	-0.12	0.04	0.22	0.29	0.09	0.03	-0.10	0.08	-0.12	0.14	0.01	0.09	0.02
4-Hydroxyproline	-0.13	-0.07	0.06	-0.18	0.11	-0.02	-0.07	0.02	0.20	0.11	0.03	0.23	0.06	0.18	0.14	0.02	-0.10	0.16	0.00	-0.19	0.05	0.11	-0.04	-0.13	-0.06	-0.16	-0.07	-0.05	0.02	-0.12
Citric acid	-0.15	0.02	0.10	-0.22	0.14	0.14	-0.06	0.11	-0.01	-0.06	0.15	-0.05	-0.13	0.01	0.12	-0.09	0.02	0.10	-0.04	-0.01	0.04	0.20	-0.11	0.04	0.11	0.08	0.17	0.08	-0.09	-0.13
Oxoglutaric acid	-0.15	-0.04	0.12	0.17	0.03	-0.03	-0.02	0.06	-0.04	0.31	0.00	0.06	0.08	0.17	-0.03	-0.05	-0.05	-0.09	0.11	0.08	-0.05	-0.11	0.15	0.10	0.10	-0.09	0.03	0.02	0.21	-0.01
Total BR	-0.17	-0.04	-0.06	-0.18	-0.04	-0.01	-0.02	-0.10	0.14	-0.05	0.09	-0.16	-0.10	0.07	-0.12	-0.02	-0.03	-0.03	-0.04	0.19	-0.07	-0.05	0.23	0.03	-0.37	-0.18	0.19	-0.06	0.10	0.09
Glycine	-0.17	-0.05	0.02	0.01	0.22	0.03	0.14	-0.12	-0.16	-0.07	0.01	0.07	0.06	0.00	0.00	-0.17	0.21	-0.15	-0.13	0.24	-0.04	-0.22	-0.08	0.03	0.13	-0.05	0.06	0.13	0.17	0.02
D-threo-Isocitric acid	-0.19	0.01	0.04	-0.06	0.19	0.11	-0.05	0.18	-0.07	0.03	0.15	-0.08	-0.21	-0.01	0.06	-0.07	-0.08	0.13	-0.09	0.17	-0.01	0.24	-0.12	-0.08	-0.01	0.08	-0.06	0.03	0.08	0.15
L-Methionine	-0.19	-0.15	-0.02	-0.17	-0.04	-0.08	-0.01	-0.01	-0.02	0.05	0.00	-0.13	0.05	0.17	0.06	-0.11	0.03	-0.04	0.03	0.00	-0.14	0.01	0.11	0.05	-0.04	0.13	-0.06	-0.14	-0.07	-0.04
L-Tyrosine	-0.23	-0.12	-0.04	-0.10	0.00	-0.04	-0.08	-0.05	0.08	0.05	-0.12	-0.15	0.14	-0.03	0.01	0.03	0.05	-0.07	0.03	-0.03	0.03	0.00	-0.05	0.11	-0.02	0.24	-0.06	0.07	0.04	-0.09
L-Aspartic acid	-0.24	-0.02	-0.05	0.16	-0.10	0.06	0.04	0.05	-0.03	-0.04	-0.05	0.04	-0.10	-0.05	-0.07	0.07	0.08	0.01	-0.09	-0.11	-0.12	0.02	0.09	-0.01	0.04	0.00	0.08	-0.06	0.09	0.05
L-Glutamic acid	-0.25	-0.02	-0.02	0.20	-0.06	-0.01	0.02	-0.03	0.02	-0.02	-0.07	0.06	-0.03	-0.02	0.03	0.06	0.03	0.06	-0.04	-0.08	0.02	0.02	0.04	0.01	-0.06	-0.01	-0.11	0.02	0.03	-0.01
L-Phenylalanine	-0.25	-0.09	-0.06	0.10	-0.05	0.01	-0.03	-0.04	0.02	0.08	0.02	-0.02	0.08	-0.01	-0.05	0.10	0.09	-0.05	-0.04	-0.07	0.02	0.00	0.02	-0.04	0.01	-0.12	0.03	0.02	0.08	-0.08

PC31	PC32	PC33	PC34	PC35	PC36	PC37	PC38	PC39	PC40	PC41	PC42	PC43	PC44	PC45	PC46	PC47	PC48	PC49	PC50	PC51	PC52	PC53	PC54	PC55	PC56	PC57	PC58	PC59	PC60	PC61	PC62
0.08	-0.05	0.03	-0.05	-0.01	0.01	-0.04	0.02	0.00	-0.01	0.03	-0.03	-0.04	0.00	0.06	0.07	0.13	-0.02	-0.02	0.04	-0.01	-0.08	0.01	0.01	0.05	-0.01	0.11	-0.20	0.12	0.08	-0.05	-0.22
0.20	-0.05	-0.09	-0.19	-0.18	-0.16	0.12	-0.20	0.05	0.05	-0.43	0.05	0.03	0.07	-0.15	-0.05	0.14	-0.06	0.05	-0.08	-0.07	0.09	-0.19	-0.13	0.06	0.19	-0.15	-0.16	-0.09	-0.09	0.04	-0.01
-0.11	0.01	-0.05	-0.08	0.04	-0.03	0.07	0.14	0.12	0.06	0.01	-0.11	-0.04	-0.07	-0.31	0.02	0.10	0.12	-0.24	-0.20	-0.05	0.04	0.02	0.10	-0.22	0.04	-0.15	0.11	-0.12	-0.11	-0.01	0.09
0.07	0.05	0.10	-0.14	0.00	0.10	-0.19	-0.01	-0.13	0.15	0.01	-0.13	0.13	-0.08	-0.12	0.07	-0.40	0.00	-0.03	0.15	-0.21	0.01	-0.19	-0.11	-0.03	-0.08	0.01	0.15	0.10	-0.04	0.27	-0.14
0.09	0.08	-0.03	0.02	0.21	-0.07	-0.01	0.17	-0.05	0.29	-0.05	0.02	0.16	-0.05	0.18	-0.07	0.03	0.05	0.02	0.18	-0.10	0.18	-0.01	-0.10	-0.04	-0.05	-0.07	0.04	-0.27	-0.03	-0.17	0.04
-0.10	0.26	0.06	0.07	-0.23	-0.09	-0.03	-0.15	-0.01	-0.11	0.03	-0.10	-0.03	-0.11	-0.06	0.00	-0.19	-0.10	-0.16	-0.01	0.08	0.09	0.05	0.04	-0.03	0.10	-0.04	0.13	0.15	0.01	-0.03	0.08
-0.15	-0.05	0.22	0.05	0.04	-0.29	0.31	-0.04	0.13	0.05	0.04	-0.06	0.05	0.04	0.09	0.06	0.13	0.29	0.07	0.12	0.05	-0.09	-0.04	-0.04	0.02	0.10	0.02	-0.10	0.01	0.06	0.23	0.03
0.03	-0.13	-0.02	0.09	0.11	0.07	-0.17	-0.02	-0.08	0.07	-0.08	-0.11	0.03	-0.03	0.04	0.00	0.15	0.04	-0.07	0.14	0.16	0.01	0.11	-0.04	0.25	0.20	-0.07	0.17	0.10	0.10	-0.04	0.04
-0.34	-0.09	0.09	0.11	0.12	-0.13	0.02	-0.10	-0.23	0.18	0.27	0.01	0.05	-0.03	-0.04	-0.04	0.08	-0.09	-0.16	-0.04	-0.07	-0.15	-0.04	0.16	-0.12	0.04	-0.08	-0.03	-0.09	-0.02	-0.03	-0.02
-0.11	-0.34	0.11	-0.13	-0.17	0.08	-0.18	0.19	-0.24	-0.07	-0.13	0.06	0.09	0.02	0.10	-0.02	0.14	0.08	-0.06	0.07	0.00	0.01	0.08	0.11	-0.11	0.04	0.04	0.04	-0.10	0.06	-0.09	0.05
-0.28	0.03	-0.06	-0.02	0.05	0.06	0.13	0.03	-0.07	0.02	0.08	-0.21	-0.06	0.17	0.11	-0.10	0.00	-0.33	0.16	-0.19	0.07	0.13	0.11	0.00	0.04	0.03	0.07	-0.04	-0.09	-0.24	0.06	0.11
-0.13	0.05	-0.06	0.05	-0.07	0.04	-0.02	-0.09	-0.08	0.03	0.08	0.08	-0.05	0.03	-0.04	0.11	0.01	0.11	0.09	0.05	-0.03	0.05	-0.12	-0.04	-0.17	0.01	0.01	0.10	-0.15	0.14	-0.12	0.18
-0.03	0.05	-0.10	-0.03	-0.07	0.10	0.14	0.11	0.09	-0.31	0.13	0.06	0.33	0.00	0.23	0.09	0.01	0.00	-0.01	0.16	0.11	-0.08	-0.02	-0.02	-0.17	0.04	-0.03	-0.10	-0.02	-0.18	0.01	0.04
0.02	-0.06	0.13	0.33	-0.05	-0.02	-0.22	0.09	0.03	-0.21	-0.05	-0.20	0.03	-0.18	-0.03	0.08	0.29	-0.05	0.17	-0.20	-0.04	0.06	-0.35	0.02	0.02	0.03	0.01	0.10	0.14	0.17	0.16	0.02
-0.12	0.11	0.05	-0.12	0.06	0.01	0.04	0.02	0.06	-0.08	-0.10	-0.06	-0.07	0.24	-0.01	0.04	-0.10	0.14	0.00	-0.04	-0.05	-0.23	0.00	-0.03	0.01	0.04	0.13	0.08	-0.04	-0.02	0.01	-0.03
0.00	-0.04	0.06	-0.07	0.05	0.10	0.09	0.00	-0.01	-0.05	0.02	-0.08	0.05	0.11	0.15	0.24	0.06	-0.06	0.11	-0.01	-0.19	-0.25	-0.06	-0.10	-0.04	0.10	-0.08	-0.08	0.08	-0.08	-0.15	-0.13
0.11	0.21	-0.08	0.21	-0.03	0.02	-0.07	0.19	0.12	0.00	0.06	0.01	0.04	0.02	0.10	0.10	-0.15	0.20	-0.04	-0.02	-0.05	0.06	0.18	-0.05	-0.02	0.14	-0.09	-0.10	0.08	0.15	-0.11	0.06
0.04	0.05	0.15	-0.13	-0.06	-0.12	0.05	0.03	-0.07	-0.06	-0.04	-0.03	-0.10	0.12	-0.09	-0.18	0.08	-0.13	0.20	-0.17	-0.12	-0.05	0.03	-0.06	-0.13	-0.26	-0.04	-0.02	0.33	0.08	-0.08	0.13
-0.18	0.05	0.11	-0.02	-0.03	0.21	0.04	-0.12	0.06	-0.15	0.00	0.01	-0.11	0.00	-0.16	0.18	-0.06	-0.16	-0.06	0.13	0.09	0.15	-0.05	-0.13	0.13	0.01	-0.04	-0.05	0.10	0.04	-0.09	-0.05
0.12	-0.04	-0.01	-0.08	-0.01	0.10	0.28	0.21	-0.11	0.02	-0.09	0.08	-0.16	-0.04	-0.16	-0.02	-0.21	-0.13	0.15	0.05	0.27	-0.16	0.07	0.02	0.17	0.03	0.22	0.05	-0.04	0.01	0.07	-0.05
-0.06	-0.09	-0.03	0.09	-0.03	0.05	0.01	0.03	0.06	-0.10	-0.11	0.03	-0.04	0.02	-0.08	-0.06	-0.05	-0.02	0.09	0.11	-0.06	-0.14	-0.02	0.06	0.00	-0.09	0.01	0.08	0.03	-0.04	-0.05	-0.21
0.05	0.07	-0.25	0.09	0.11	-0.01	0.09	-0.10	0.02	-0.04	-0.08	0.03	0.03	-0.01	-0.06	0.02	-0.03	-0.15	-0.26	0.12	0.01	-0.15	-0.12	0.10	0.10	0.00	0.22	-0.08	0.00	0.31	-0.36	0.00
-0.03	0.16	-0.12	0.04	-0.04	-0.16	0.02	0.15	-0.03	0.04	-0.15	0.02	-0.10	0.15	0.09	0.09	0.02	-0.10	0.15	0.30	-0.03	0.02	-0.08	0.20	-0.12	-0.23	-0.20	0.00	-0.04	0.09	0.11	-0.17
0.03	-0.01	0.04	0.11	0.00	-0.01	-0.25	0.00	0.16	-0.08	0.01	0.21	0.00	0.12	-0.01	0.01	-0.03	-0.10	0.07	-0.04	-0.11	-0.08	0.05	-0.07	0.00	-0.10	-0.21	0.06	-0.01	-0.06	-0.07	0.11
0.11	-0.10	-0.07	0.01	-0.04	0.19	-0.07	-0.01	-0.02	0.14	-0.14	-0.06	0.02	0.16	0.00	0.07	-0.11	0.05	-0.04	0.02	0.09	0.07	0.00	0.24	-0.23	-0.06	-0.04	-0.15	-0.04	0.20	0.03	0.05
0.00	0.07	0.04	0.24	0.04	0.06	-0.09	-0.18	0.04	-0.03	-0.10	0.01	-0.03	-0.09	0.05	0.12	-0.16	0.10	0.02	-0.02	-0.03	0.04	-0.10	-0.05	-0.06	-0.02	0.17	-0.15	-0.28	-0.26	-0.03	-0.01
0.24	0.04	-0.10	0.14	-0.08	0.14	0.06	0.05	-0.29	-0.02	0.02	0.12	-0.14	0.11	0.00	-0.01	0.10	0.05	0.08	0.09	0.05	-0.10	0.02	-0.15	-0.14	0.11	-0.12	0.00	0.05	-0.30	0.00	0.03
-0.10	0.02	0.11	0.01	0.24	-0.05	0.01	0.28	0.20	-0.11	0.04	0.12	0.02	0.11	-0.08	-0.04	-0.07	-0.06	0.09	-0.03	0.00	-0.06	0.01	-0.05	0.16	-0.04	-0.10	-0.14	0.00	-0.02	0.02	-0.05
0.21	0.00	-0.03	-0.02	0.09	-0.01	0.05	0.04	0.06	-0.11	0.19	0.15	0.22	0.21	-0.15	-0.10	0.09	0.02	0.00	0.02	-0.15	0.04	-0.15	0.24	0.24	-0.07	0.01	0.12	0.01	-0.09	-0.07	0.14
-0.09	0.24	-0.25	-0.14	-0.13	-0.21	-0.09	0.15	0.13	0.00	0.06	0.07	-0.07	-0.17	0.06	0.02	0.02	0.05	0.07	0.13	-0.01	-0.02	-0.04	-0.07	0.11	0.15	0.03	0.11	-0.03	-0.03	0.10	0.08
-0.08	0.06	0.10	0.21	-0.05	0.15	0.39	-0.14	-0.09	-0.02	-0.10	0.19	0.02	-0.04	0.05	-0.06	-0.02	0.16	-0.07	-0.22	0.00	0.23	-0.09	0.08	0.02	-0.05	0.09	-0.01	0.08	0.00	-0.13	-0.08
-0.06	-0.08	-0.02	-0.08	0.22	0.13	0.03	0.08	0.20	-0.13	-0.10	0.04	0.06	0.16	0.06	-0.19	-0.02	-0.03	-0.20	0.00	0.07	0.15	-0.11	0.03	-0.20	0.12	0.19	0.22	-0.01	0.01	0.22	0.02
-0.02	0.12	-0.19	0.11	0.07	-0.01	0.04	-0.05	-0.05	-0.02	0.08	-0.06	0.14	0.11	-0.15	-0.04	0.24	0.01	0.18	0.13	-0.05	-0.17	-0.03	0.01	-0.02	-0.08	0.17	0.18	0.03	-0.03	0.00	0.07
-0.08	0.06	-0.09	-0.04	-0.09	-0.07	0.20	0.09	-0.13	0.03	-0.04	0.15	0.09	-0.10	0.16	-0.08	-0.13	-0.03	-0.18	-0.01	-0.19	-0.03	0.02	0.09	0.18	0.03	-0.17	0.09	0.14	0.14	0.25	-0.12
-0.01	0.02	0.11	-0.06	0.07	0.01	-0.05	0.08	-0.21	-0.09	-0.01	-0.08	-0.17	-0.01	0.05	0.07	-0.08	0.08	0.25	-0.03	-0.31	0.06	0.07	0.22	0.14	0.21	0.15	-0.03	-0.09	-0.05	-0.23	0.06
-0.08	0.22	0.22	-0.10	-0.02	-0.12	-0.17	-0.04	-0.09	-0.02	-0.04	0.25	-0.10	0.15	0.13	0.11	0.08	0.18	0.00	0.06	0.34	-0.01	-0.20	0.07	0.05	0.02	0.03	0.10	0.09	-0.06	-0.08	0.19
0.11	0.08	0.12	0.01	0.01	-0.03	0.00	0.00	0.10	0.13	-0.13	0.03	0.11	0.02	0.02	-0.14	0.13	-0.15	-0.09	0.03	-0.16	-0.05	0.09	0.07	-0.07	-0.18	0.19	-0.14	-0.01	0.16	0.04	0.18

0.08	0.02	0.04	-0.21	-0.01	0.26	0.15	-0.06	0.15	0.13	0.23	0.13	0.19	-0.26	-0.19	0.07	0.12	-0.07	0.28	-0.02	0.17	0.00	0.01	-0.02	-0.13	-0.03	-0.05	0.04	-0.07	0.09	-0.03	0.01
0.00	-0.09	-0.04	-0.14	-0.09	-0.15	0.01	-0.07	-0.12	-0.06	0.19	0.08	-0.11	0.07	0.03	-0.01	-0.09	0.24	-0.08	-0.02	0.03	-0.03	0.03	-0.30	-0.02	-0.21	0.08	0.00	0.12	0.19	0.00	0.16
0.19	-0.13	0.12	0.15	-0.01	-0.18	-0.03	-0.05	-0.02	0.07	0.05	-0.07	-0.08	0.12	0.02	-0.06	-0.10	-0.08	0.10	0.01	-0.06	-0.11	0.18	-0.13	-0.08	0.08	0.09	0.21	-0.11	0.00	0.00	-0.04
-0.07	-0.10	-0.08	0.08	-0.04	-0.05	-0.04	0.01	-0.15	-0.09	0.00	-0.22	0.21	0.24	-0.06	-0.16	-0.15	-0.07	-0.15	0.13	0.14	-0.01	-0.34	-0.17	0.08	-0.14	-0.12	-0.18	0.02	-0.17	-0.09	0.13
-0.09	0.02	0.22	-0.18	0.00	0.08	-0.12	-0.14	0.10	0.06	-0.07	-0.09	0.05	0.03	0.06	-0.06	0.04	0.15	0.00	0.09	-0.06	0.18	0.11	0.13	0.17	-0.24	-0.09	-0.09	-0.03	-0.10	0.03	-0.13
0.19	-0.11	-0.11	0.10	-0.05	-0.12	0.17	-0.05	0.13	0.11	0.13	-0.18	-0.24	0.01	0.01	0.01	0.03	-0.14	-0.11	0.08	-0.04	0.06	-0.20	0.11	0.06	0.08	-0.10	0.21	-0.01	-0.03	-0.02	0.04
0.02	0.01	-0.05	-0.03	0.04	0.13	-0.12	-0.27	0.23	-0.02	0.21	-0.09	-0.10	0.18	-0.15	-0.11	-0.02	0.14	-0.01	0.11	-0.04	-0.13	0.15	0.20	0.08	0.24	-0.24	-0.20	0.10	0.01	0.03	0.00
0.02	-0.07	-0.14	0.01	0.27	-0.05	0.10	0.22	0.07	0.05	-0.16	-0.10	-0.21	0.01	-0.07	0.19	0.05	0.25	-0.01	-0.14	0.11	0.17	-0.02	-0.03	0.05	-0.20	-0.11	-0.07	0.16	-0.08	-0.12	-0.02
-0.01	0.10	0.23	0.06	0.02	-0.09	0.15	-0.14	0.11	0.03	-0.26	-0.13	0.05	0.01	0.06	-0.03	-0.09	-0.03	0.06	0.07	0.18	-0.24	0.04	-0.03	-0.26	0.08	-0.16	0.14	-0.03	0.13	-0.17	-0.02
0.06	0.17	-0.07	-0.02	0.08	-0.07	0.01	-0.02	-0.09	0.09	0.16	-0.07	0.13	0.06	0.01	0.05	0.20	0.07	0.04	-0.02	0.03	0.07	0.00	-0.02	-0.04	0.07	0.10	0.00	0.06	0.06	0.01	-0.26
0.05	0.16	0.07	0.12	-0.06	0.18	0.02	0.16	-0.08	0.24	0.10	-0.18	-0.06	-0.04	0.01	0.10	0.15	-0.12	-0.10	0.14	0.23	0.01	0.07	-0.01	-0.01	-0.16	-0.09	-0.11	0.11	-0.09	0.13	0.07
-0.13	-0.31	-0.33	-0.07	-0.30	-0.06	-0.08	-0.01	0.11	0.20	-0.07	0.11	0.05	0.08	0.02	0.11	-0.06	0.04	0.12	-0.09	0.07	0.04	0.00	0.04	0.01	0.10	0.02	0.01	0.11	-0.07	-0.02	0.06
0.14	0.08	0.07	0.10	0.01	-0.02	-0.01	-0.04	0.01	0.05	-0.11	0.01	0.01	-0.15	-0.09	-0.21	0.07	0.18	0.05	0.13	0.07	-0.15	-0.08	0.08	0.08	0.11	0.04	-0.26	-0.05	-0.09	0.16	0.07
0.03	0.07	-0.11	-0.03	-0.27	-0.14	-0.11	0.02	0.01	-0.20	0.07	-0.11	-0.16	-0.06	-0.06	-0.10	0.20	0.01	-0.18	0.01	0.01	-0.02	0.15	0.01	-0.20	-0.13	0.20	-0.16	-0.12	-0.09	-0.01	-0.29
-0.20	-0.02	-0.25	0.19	0.08	-0.08	0.02	0.03	0.08	-0.05	-0.12	-0.11	0.06	0.01	0.07	-0.04	0.00	-0.01	0.01	-0.02	-0.03	0.00	0.24	-0.12	0.06	-0.05	0.04	-0.04	0.00	0.03	0.05	0.09
0.06	-0.18	0.16	0.10	-0.10	-0.03	0.16	-0.03	0.04	0.01	0.05	-0.03	-0.08	0.15	0.18	0.30	0.03	-0.08	-0.12	0.10	-0.02	-0.06	0.03	0.08	0.16	-0.08	0.01	0.02	-0.08	0.03	0.13	-0.02
0.11	-0.02	0.03	-0.13	-0.20	-0.16	0.14	0.08	-0.02	-0.23	0.18	-0.22	0.08	0.13	0.03	0.08	-0.12	-0.01	0.19	0.06	0.07	0.30	-0.04	0.23	-0.02	0.05	-0.06	-0.07	-0.17	0.19	-0.05	0.03
-0.04	-0.28	-0.06	0.02	0.06	-0.02	0.06	-0.21	0.01	-0.01	0.01	-0.21	0.18	-0.22	0.09	0.01	-0.01	0.07	0.18	0.12	0.00	0.04	0.07	-0.18	0.11	-0.09	0.05	-0.01	0.04	0.06	0.00	0.11
0.00	-0.08	-0.14	0.07	0.20	0.08	0.00	-0.23	-0.13	-0.14	-0.03	0.24	-0.11	0.14	0.02	0.14	0.06	0.03	0.03	0.01	-0.06	0.05	0.02	0.09	-0.14	0.01	0.05	0.00	0.02	0.02	0.42	0.00
-0.03	-0.14	0.03	0.11	-0.08	0.23	-0.07	0.17	0.20	-0.10	-0.07	0.00	-0.04	-0.06	0.14	-0.10	-0.02	0.02	0.05	0.12	-0.02	-0.05	-0.12	0.09	-0.15	0.02	0.07	0.05	0.17	0.04	-0.07	0.01
-0.05	0.00	0.05	0.03	-0.06	-0.04	-0.07	-0.07	0.01	0.01	-0.11	0.14	0.08	0.04	0.09	-0.17	0.03	-0.16	0.03	-0.06	0.16	-0.05	0.18	0.09	0.08	0.00	-0.21	0.07	-0.12	0.04	0.01	0.10
-0.11	0.08	0.04	-0.08	-0.20	0.17	0.07	0.00	0.13	0.05	-0.09	-0.22	-0.07	0.07	0.12	-0.05	0.18	0.04	-0.08	0.05	-0.11	-0.04	-0.07	-0.04	0.04	0.14	0.05	0.13	0.01	-0.12	-0.01	0.01
0.13	0.09	0.10	-0.10	0.05	0.08	0.06	0.19	-0.02	-0.22	-0.06	-0.06	0.02	-0.11	0.13	-0.10	0.01	-0.04	-0.11	-0.07	-0.10	0.02	0.06	-0.16	-0.01	0.18	-0.07	-0.10	-0.02	0.07	0.10	0.15
0.12	-0.08	0.11	-0.10	-0.05	-0.16	0.07	-0.03	0.17	-0.07	-0.05	0.06	0.19	-0.11	-0.03	0.30	0.04	-0.02	-0.21	0.05	-0.11	-0.05	0.12	0.05	-0.03	-0.16	0.06	0.19	0.11	-0.33	-0.13	0.04
-0.02	-0.06	0.02	-0.09	0.00	0.15	0.06	-0.01	0.18	0.21	0.17	0.09	-0.35	-0.04	0.30	-0.18	0.02	-0.03	-0.02	0.05	-0.16	-0.03	-0.29	-0.10	-0.05	-0.05	-0.03	0.01	-0.03	0.01	-0.10	0.06
0.13	0.14	0.02	0.05	-0.05	0.02	-0.02	-0.12	0.06	-0.04	0.04	-0.08	0.02	0.14	0.05	-0.11	-0.07	0.10	0.04	-0.08	0.00	0.09	-0.09	-0.12	0.04	-0.14	0.12	0.08	-0.21	0.05	0.07	0.02
-0.05	0.15	-0.22	-0.30	0.19	0.11	0.00	-0.21	-0.17	-0.15	-0.11	-0.10	0.01	-0.04	0.17	0.09	0.14	-0.05	-0.01	-0.07	-0.06	-0.07	-0.07	-0.09	-0.05	-0.03	-0.22	0.02	-0.03	0.14	-0.05	-0.07
-0.20	0.07	0.05	-0.04	-0.03	-0.04	0.05	0.00	-0.06	0.14	-0.10	0.07	0.04	0.04	-0.11	-0.13	0.05	-0.02	0.08	0.35	-0.17	0.23	0.05	0.00	-0.07	0.15	0.21	-0.13	0.32	-0.08	-0.08	0.08
0.16	0.00	0.10	-0.01	0.14	-0.21	-0.13	-0.02	0.02	0.15	0.08	0.12	0.12	0.14	0.14	0.13	-0.01	-0.29	-0.08	-0.16	0.09	0.18	-0.06	-0.09	-0.12	0.29	0.12	-0.15	0.17	-0.03	-0.03	-0.14
0.06	-0.21	0.06	-0.06	0.20	-0.21	0.04	0.03	-0.17	-0.23	0.07	0.01	-0.14	-0.27	-0.08	-0.18	-0.07	0.02	-0.07	0.17	0.07	-0.01	-0.10	0.07	-0.13	0.08	-0.14	-0.06	0.00	-0.07	-0.06	-0.01
0.14	0.10	-0.02	-0.13	0.09	0.08	-0.01	0.00	-0.20	0.03	-0.12	-0.23	0.04	0.03	0.03	0.11	0.03	0.05	-0.12	-0.08	0.04	-0.12	0.06	0.20	0.08	0.02	0.07	0.13	0.04	-0.08	0.03	0.27
-0.17	0.03	0.02	-0.13	-0.02	-0.06	-0.20	0.11	0.03	0.08	-0.03	0.02	0.04	-0.06	0.01	0.00	0.04	-0.04	0.00	-0.13	0.18	-0.18	-0.22	0.07	0.20	-0.03	0.11	-0.04	-0.18	0.02	-0.02	-0.18
-0.01	-0.01	0.01	0.09	-0.23	0.08	0.11	0.16	-0.12	0.16	0.08	-0.03	0.22	0.11	-0.03	-0.07	-0.08	0.20	-0.03	-0.27	-0.10	-0.23	-0.11	0.00	0.00	0.05	-0.07	-0.09	0.06	0.06	-0.01	-0.08
-0.05	0.07	-0.07	0.11	0.09	-0.08	-0.02	-0.05	0.00	0.01	-0.08	-0.06	0.11	-0.03	0.04	-0.13	-0.13	0.10	0.20	-0.05	0.08	0.06	-0.01	0.15	-0.10	0.00	-0.09	0.22	0.18	-0.14	-0.10	-0.28
-0.11	0.09	0.00	0.18	-0.11	0.00	0.04	0.00	-0.09	-0.04	-0.09	0.16	0.00	-0.17	-0.07	0.19	0.03	-0.17	0.03	0.02	-0.21	-0.02	-0.04	0.05	0.08	-0.04	-0.12	-0.10	-0.11	-0.02	0.04	0.14
-0.15	-0.02	0.02	-0.06	0.09	-0.07	-0.02	0.12	0.07	0.08	-0.08	-0.10	-0.02	0.04	-0.25	0.25	-0.12	-0.03	0.05	0.04	-0.08	-0.09	-0.11	-0.03	-0.20	0.11	0.07	-0.12	-0.05	0.09	0.09	0.19
-0.08	-0.02	0.13	0.08	-0.06	0.09	0.05	0.13	-0.08	-0.04	-0.02	0.07	0.02	0.23	-0.30	0.02	0.19	0.05	-0.11	0.16	-0.06	0.17	0.08	-0.31	0.08	0.12	-0.11	0.18	-0.20	0.15	-0.05	-0.21

Supplementary Table S6. PCA variance when using the ouctomes selected by the visual extraction rule

	Individual	Cumulative
PC1	**0.43**	0.43
PC2	**0.29**	0.72
PC3	0.21	0.93
PC4	0.07	1.00

Supplementary Table S7. PCA loadings when using outcomes selected by Visual Extraction Algorithm

	PC1	PC2	PC3	PC4
L-Glutamic acid	0.69	0.13	-0.26	-0.67
Taurine	-0.01	0.81	-0.48	0.33
L-Asparagine	-0.32	-0.45	-0.83	-0.10
BCAA/AAA	-0.65	0.35	0.14	-0.66
